# An autoregulatory poison exon in *Smndc1* is conserved across kingdoms and influences organism growth

**DOI:** 10.1371/journal.pgen.1011363

**Published:** 2024-08-16

**Authors:** Andrea E. Belleville, James D. Thomas, Jackson Tonnies, Austin M. Gabel, Andrea Borrero Rossi, Priti Singh, Christine Queitsch, Robert K. Bradley

**Affiliations:** 1 Computational Biology Program, Public Health Sciences Division, Fred Hutchinson Cancer Center, Seattle, Washington, United States of America; 2 Basic Sciences Division, Fred Hutchinson Cancer Center, Seattle, Washington, United States of America; 3 Molecular and Cellular Biology Graduate Program, University of Washington, Seattle, Washington, United States of America; 4 Medical Scientist Training Program, University of Washington, Seattle, Washington, United States of America; 5 Department of Genome Sciences, University of Washington, Seattle, Washington, United States of America; 6 Graduate Program in Biology, University of Washington, Seattle, Washington, United States of America; 7 Preclinical Modeling Core, Fred Hutchinson Cancer Center, Seattle, Washington, United States of America; 8 Brotman Baty Institute for Precision Medicine, Seattle, Washington, United States of America; The University of North Carolina at Chapel Hill, UNITED STATES OF AMERICA

## Abstract

Many of the most highly conserved elements in the human genome are “poison exons,” alternatively spliced exons that contain premature termination codons and permit post-transcriptional regulation of mRNA abundance through induction of nonsense-mediated mRNA decay (NMD). Poison exons are widely assumed to be highly conserved due to their presumed importance for organismal fitness, but this functional importance has never been tested in the context of a whole organism. Here, we report that a poison exon in *Smndc1* is conserved across mammals and plants and plays a molecular autoregulatory function in both kingdoms. We generated mouse and *A*. *thaliana* models lacking this poison exon to find its loss leads to deregulation of SMNDC1 protein levels, pervasive alterations in mRNA processing, and organismal size restriction. Together, these models demonstrate the importance of poison exons for both molecular and organismal phenotypes that likely explain their extraordinary conservation.

## Author summary

Poison exons are highly conserved elements in the human genome and are known for their roles in regulating mRNA abundance. Despite their assumed importance for organismal fitness, their functional significance has not been tested in whole organisms. Our study focuses on a poison exon in the *Smndc1* gene. We discovered that this poison exon is conserved between mammals and plants and sought to test its functional importance. We generated mouse and plant models lacking this specific poison exon. The loss of this exon led to dysregulated SMNDC1 protein levels and widespread disruptions in RNA processing. These molecular changes resulted in a noticeable reduction in organismal size, underscoring the physiological impact of poison exon loss. Our findings demonstrate that poison exons are crucial for maintaining proper protein levels and mRNA processing homeostasis, which may partially explain their remarkable evolutionary conservation. This study highlights the importance of poison exons in both molecular regulation and overall organismal health, providing insights into their conservation across diverse species.

## Introduction

Alternative splicing (AS) is a post-transcriptional mechanism that regulates the function and expression of multi-exonic genes. While AS expands an organism’s proteomic diversity, it is estimated that up to one third of human genes produce non-coding mRNAs which are immediately subject to degradation [1–3]. Specifically, inclusion of a class of cassette exons termed ‘poison exons’ introduces one or more premature termination codons (PTC) into the mature mRNA, subjecting the mRNA transcript to degradation via nonsense-mediated mRNA decay (NMD) [4]. Poison exon AS coupled to NMD serves as a means of post-transcriptional gene regulation, and it has been implicated as a critical mechanism in neurodevelopment, environmental adaptation, and disease [5–9].

AS of poison exons is a conserved regulatory process across metazoa, yeast, and plants [10–12]. Many poison exons exhibit significant nucleotide sequence conservation, despite lacking protein coding potential, and conserved poison exons are enriched in RNA-binding proteins, such as serine-arginine-rich (SR) proteins and heterogeneous nuclear ribonucleoproteins [13,14]. Within these splicing factor families, it is proposed that poison exons may provide a context-specific auto- and cross-regulatory mechanism to regulate splicing factor abundance [15–19]. Therefore, poison exon conservation within splicing factors is believed to be due to their important role in maintaining splicing factor homeostasis and consequently global splicing.

Outside of their role in regulating splicing factor abundance, little is known about the strong purifying selection driving poison exon sequence conservation. Our recent work demonstrated that endogenous deletion of poison exons influenced growth rates in cancer cell lines, an indication of their potential impact on cellular fitness [8]. Beyond this, no study has yet tested the essentiality of poison exons in an *in vivo* setting. This underlines the pressing need for further investigation into their functional relevance in organismal physiology.

In this study, we undertook a detailed study of a poison exon that is present in both mammalian and plant genomes despite an evolutionary distance exceeding 1 billion years [20]. Specifically, we studied the poison exon within the gene Survival motor neuron domain containing 1 (*Smndc1*), which encodes a well-conserved spliceosome component SMNDC1. SMNDC1 contains a Tudor domain by which it binds methylated arginines on other splicing proteins, playing an essential role in spliceosome assembly [21–24]. Like many other genes encoding splicing factors, *SMNDC1* contains an NMD-targeted poison exon which is thought to regulate *SMNDC1* transcript abundance [25,26]. Recently, an ortholog to *SMNDC1* was identified in plants called splicing factor 30 (*SPF30*), which both regulates AS [27] and undergoes extensive AS in many plant species [28]. Given the conservation of *SMNDC1* and *SPF30* across kingdoms, we hypothesized that this splicing factor gene might prove a compelling exemplar to investigate the potential essentiality of poison exons in physiological contexts.

## Results

### Identification of *SMNDC1* orthologs and unannotated poison exons

**Fig 1:**
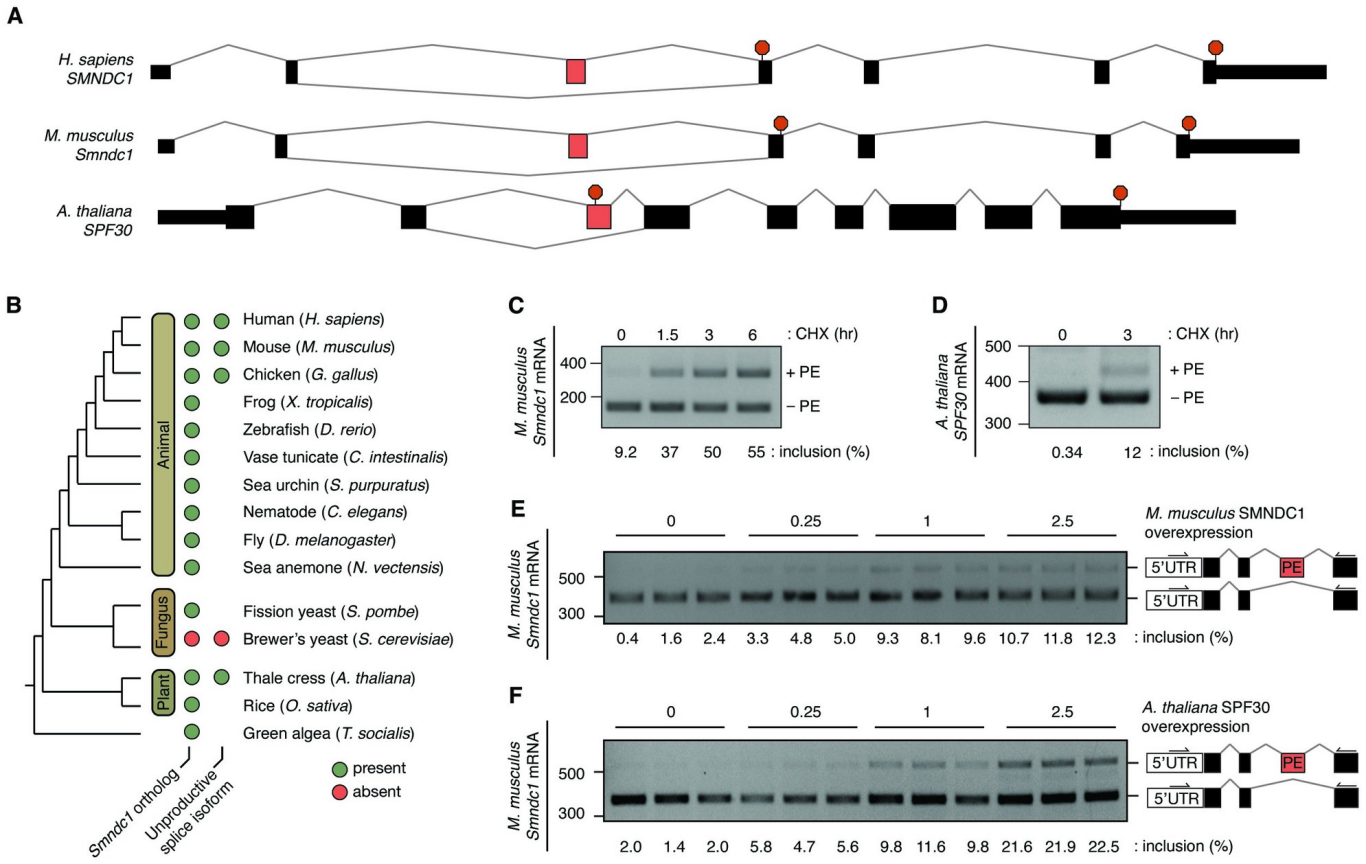
A poison exon in *Smndc1* is functionally conserved between mammals and plants. **A**, Schematic demonstrating *SMNDC1* and *Smndc1* exon structure in humans and mice, respectively, and *SPF30* exon structure in *A*. *thaliana*. Black boxes indicate coding exons, and the alternatively spliced poison exon is highlighted in red in each gene structure. Termination codons indicated by red octagons. **B**, Unscaled phylogenetic tree, indicating species with the presence or absence of identified orthologs of *Smndc1* and *SPF30* (left circles) and unproductive spliced RNA isoforms (right circles). No circle indicates insufficient data to draw a conclusion regarding presence or absence of an unproductive splice isoform. **C**, RT-PCR analysis of *Smndc1* poison exon (PE) inclusion in mouse Melan-A cells following nonsense-mediated mRNA decay (NMD) inhibition with cycloheximide (CHX) for increasing amounts of time (hours). Inclusion of the PE-containing transcript is calculated as a percentage of total transcript abundance. **D**, as in (**C**) but treatment of *A*. *thaliana* Col-0 seedlings and analysis of the *SPF30* PE inclusion following CHX treatment. **E**, RT-PCR analysis of *Smndc1* PE inclusion in mouse cells with increasing overexpression of mouse SMNDC1. PCR was performed with primers within the 5’UTR and third coding exon to capture endogenous transcript. *n* = 3 technical replicates per condition. **F**, as in (**E**) but with increasing overexpression of *A*. *thaliana* SPF30.

We first sought to understand the extent of similarity between the structures and sequences of the human *SMNDC1* and mouse *Smndc1* genes. In both human and mouse orthologs, the poison exon is located between the second and third coding exons and introduces a frameshift when spliced into the mRNA transcript and consequently introduces a PTC in the downstream coding exon (Fig 1A). At the nucleotide level, the poison exon retains greater sequence similarity in vertebrates (94.7% similarity in humans and mice), than the upstream and downstream coding exons (86.7% and 90.9%, respectively; S1A and S1B Fig).

To examine the evolutionary history of SMNDC1, we identified SMNDC1 protein orthologs using protein sequence conservation and reciprocal best hit analysis in many commonly studied animal, fungal, and plant model species (Fig 1B and S1 Table). Murine SMNDC1 shares 99.58% protein identity with the human ortholog and 30.07% identity with *A*. *thaliana* SPF30. Consistent with previously published reports [29], we did not find evidence of *SMNDC1* orthologs in fungal species with reduced genomic intron content, such as *S*. *cerevisiae*, suggesting the loss of those protein orthologs, but identified *SMNDC1* orthologs in all other queried species (Fig 1B).

We next tested whether the *Smndc1* poison exon was conserved outside of the human and mouse genomes. We first searched for unproductive splice isoforms of each orthologous gene using publicly available expressed sequence tags (ESTs). We identified a poison exon-containing isoform in *G*. *gallus* from EST data but were unable to conclusively determine whether similar unproductive splice isoforms were present in *Smndc1* orthologs in other genomes due to the relative paucity of EST data from those species (Fig 1B). Although many RNA-sequencing (RNA-seq) datasets now exist across vertebrate and fungal species, few such datasets have been produced with the high read coverage depth or in the context of NMD inhibition which are necessary for robust detection of poison exon-containing transcripts, hindering our ability to determine whether *Smndc1* unproductive isoforms exist in those model organisms.

In contrast, RNA-seq data from NMD-inhibited (cycloheximide (CHX)-treated) *A*. *thaliana* was publicly available [30]. We therefore searched these data for evidence of unproductive splicing of *SPF30*, the ortholog of *SMNDC1*. This analysis revealed an accumulation of poison exon-containing mRNA transcripts in the setting of CHX treatment and thereby NMD inhibition (S1C Fig). This NMD-targeted exon corresponded to recent evidence of AS in many *SPF30* plant orthologs [28]. Indeed, both lines of evidence suggest a previously unannotated NMD-targeted poison exon in *A*. *thaliana* SPF30.

We next sought to determine if *SMNDC1* is unique in containing a putative poison exon in both plants and mammalian orthologs. In addition to *SMNDC1*, genes encoding other core spliceosome components in humans are reported to contain NMD-targeted transcripts [25]. We identified plant orthologs to these human spliceosome components and mapped NMD-inhibited RNA-seq reads from the same *A*. *thaliana* dataset [30]. Only the ortholog to *Smndc1* demonstrated evidence of poison exon-containing RNA-seq reads in *A*. *thaliana*, suggesting that *SMNDC1*’s poison exon is particularly well conserved (S2A–S2E Fig).

To further experimentally confirm that the putative poison exons in both mouse *Smndc1* and *A*. *thaliana SPF30* are NMD-sensitizing, we treated a murine melanocyte cell line and *A*. *thaliana* seedlings with CHX. Subsequent RT-PCR demonstrated high levels of inclusion of the poison exon in both mouse and plant (Fig 1C and 1D). These data indicate that an alternatively spliced exon that triggers NMD lies in the same intronic position of the human, mouse, and *A*. *thaliana* genes (Fig 1A). Overall, we were able to conclusively identify SMNDC1 orthologs in most queried species and poison exon-containing isoforms in human, mouse, *G*. *gallus*, and *A*. *thaliana*.

### A conserved regulatory role of the *Smndc1* poison exon

Poison exons are believed to play a role in controlling RNA splicing factor abundance by targeting mRNA transcripts for degradation. This was previously reported for *SMNDC1*, where overexpression of SMNDC1 protein led to increased inclusion of the poison exon *in vitro* [25]. We therefore tested whether this role is conserved in mice and plant orthologs. We transiently overexpressed murine SMNDC1 protein in murine cells (S3A Fig) and then treated with an NMD inhibitor to allow for accumulation of poison exon-included mRNA transcripts. Using RT-PCR, we determined the degree of poison exon inclusion at different levels of SMNDC1 protein overexpression. Increased expression of SMNDC1 led to more than a ten-fold increase in the poison exon-containing transcript in mouse cells (Fig 1E).

Building on these findings, we sought to determine whether the *Smndc1* poison exon maintains a conserved regulatory role in the presence of orthologous SPF30 protein. We ectopically expressed *A*. *thaliana* SPF30 in murine cell culture and performed RT-PCR to amplify endogenous *Smndc1* mRNA transcripts (Fig 1F). Similarly to the overexpression of murine SMNDC1, the overexpression of SPF30 led to increased poison exon inclusion in mouse cells. This result is consistent with a conserved autoregulatory cycle which increases inclusion of the *Smndc1* poison exon in the context of high protein abundance. Across highly divergent species, this may be a conserved mechanism of regulating total SMNDC1 protein abundance.

### Generation of a mouse model lacking the *Smndc1* poison exon

**Fig 2:**
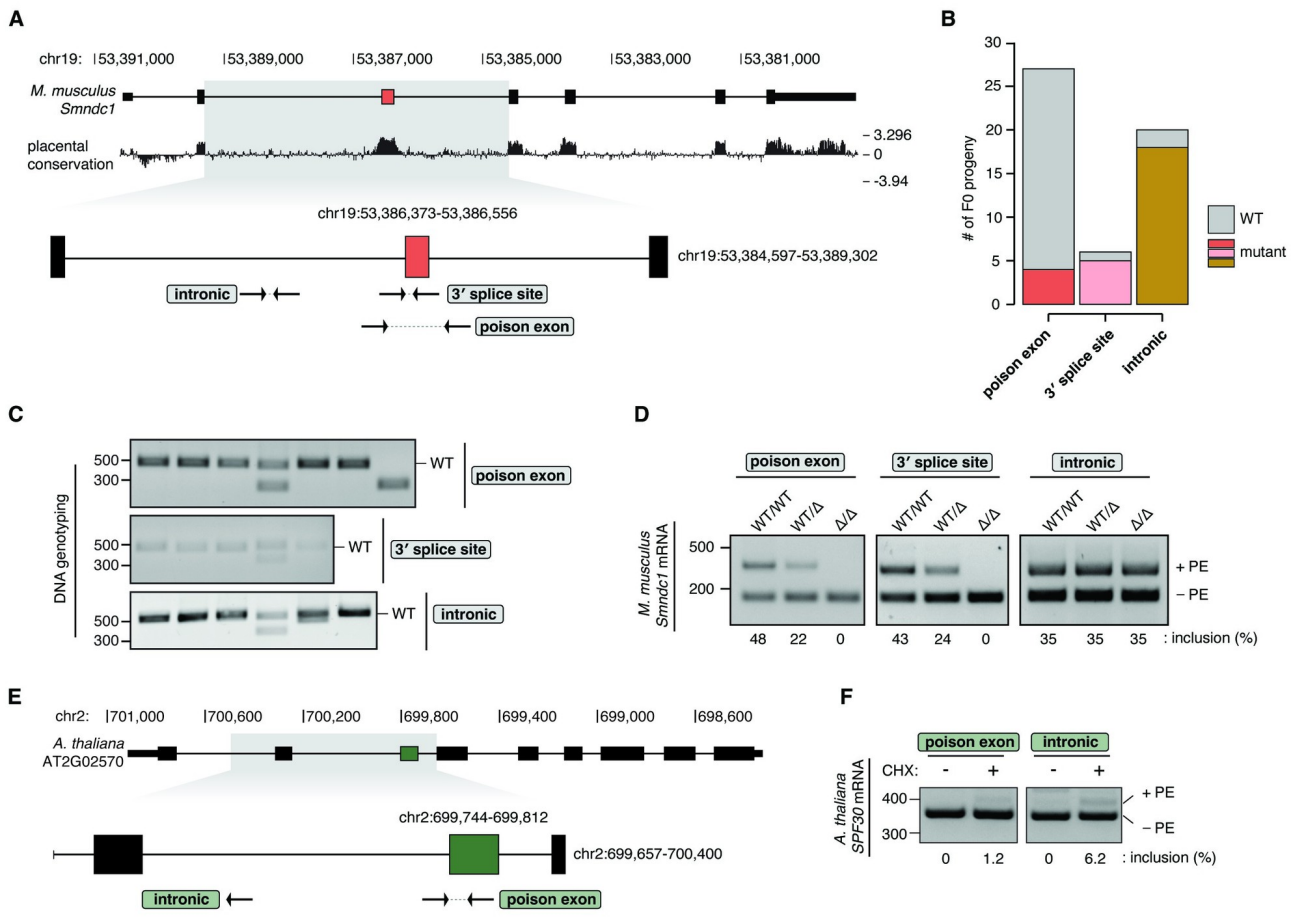
Generation of parallel poison exon null models in mouse and *A*. *thaliana*. **A**, Paired guide RNAs (pgRNA, arrows) designed to disrupt the *Smndc1* poison exon (PE) (red box). pgRNA-directed editing produced three mouse lines with deletion of the PE, the PE 3′ splice site, or upstream intronic region. Placental conservation from Placental Mammal Basewise Conservation by PhyloP (UCSC). **B**, Quantification of efficient genomic DNA (gDNA) targeting of founder mice (F0 progeny) from the three pgRNA targeting strategies designated in (**A**). PCR amplicon sequencing was used to determine gDNA targeting. **C**, Genotyping gels demonstrating DNA edits in a selection of founder mice from the three targeting strategies designated in (**A**). Wild-type (WT) band labeled. **D**, RT-PCR analysis of the *Smndc1* PE inclusion from primary mouse fibroblast lines generated from backcrossed littermates. Littermates were either wild-type (WT/WT), heterozygous (WT/Δ), or homozygous (Δ/Δ) for the respective edited alleles for each of the targeting strategies designated in (**A**). Fibroblasts were treated with cycloheximide (CHX) to inhibit NMD. Inclusion values represent inclusion of the PE transcript as a percentage of total transcript quantity. **E**, pgRNAs designed to disrupt the *A*. *thaliana SPF30* PE (green box). pgRNA-directed editing produced two *A*. *thaliana* lines with deletion of the poison exon (#1), or upstream intronic region (#2). **F**, RT-PCR analysis of PE inclusion from heterogeneous pooled populations of *A*. *thaliana* seedlings from both lines designated in (**E**). Seedlings were treated with CHX to inhibit NMD. Inclusion values represent inclusion of the PE transcript as a percentage of total transcript quantity. The faint RT-PCR band corresponding to the *SPF30* PE represents RNA transcripts remaining in the heterozygous plants and does not arise from plants homozygous for the *SPF30* PE deletion.

Given the conservation of SMNDC1 orthologs and the poison exon, we next aimed to test the essentiality of the *Smndc1* poison exon as a regulatory element *in vivo* by generating a knockout mouse model. The murine *Smndc1* locus is composed of seven NCBI RefSeq annotated exons, five constitutive exons and two alternative 5′ exons encoding untranslated regions of the mRNA (Fig 2A). Across placental vertebrates, the *Smndc1* poison exon shows higher genomic DNA conservation compared to all *Smndc1* coding cassette exons. This higher conservation is evidenced by the highest basewise phyloP conservation scores observed at the poison exon (Fig 2A). We previously demonstrated that paired guide RNA (pgRNA) targeting of 3′ splice sites minimally impacts genomic DNA sequence while efficiently abrogating exon inclusion *in vitro* [8]. We therefore selected flanking pgRNAs with optimal off-target scores to generate an allele with deletion of the *Smndc1* poison exon and an allele with isolated deletion of the poison exon 3′ splice site (S2 Table and Fig 2A). Both pairs of *Smndc1* poison exon-targeting guides fall within the locus surrounding the poison exon, which is highly conserved across placental species (S4A Fig). Lastly, to control for unanticipated effects of local editing, we generated a negative control allele with a deletion in the upstream intronic region, outside of the highly conserved region flanking the *Smndc1* poison exon (Fig 2A).

To perform CRISPR/Cas9 editing, we injected pgRNAs and Cas9 protein into the pronucleus of one-cell mouse embryos, which were then transferred to surrogate mothers. Viable pups were derived from all three targeting strategies, with successful generation of 27, 6, and 20 founder pups from the whole poison exon deletion line, 3′ splice site deletion line, and intronic control line, respectively (Fig 2B). Subsequent genotyping was carried out using PCR and Sanger sequencing, indicating many mutated founder mice (Fig 2B and 2C). From each targeting strategy, individual founder mice were selected based on sequencing results and subsequently backcrossed for four generations (S4B Fig).

To test whether pgRNA-directed CRISPR/Cas9 editing generated alleles that impacted the *Smndc1* poison exon inclusion, we generated primary fibroblast lines from wild-type, heterozygous, and homozygous littermates of each of the three deletion lines. We treated fibroblasts with CHX to inhibit NMD, as the measured inclusion of the poison exon is artificially low in steady-state cells. Using RT-PCR, we observed complete loss of the *Smndc1* poison exon-containing transcripts in mice lacking the entire poison exon (Fig 2D). Similarly, a 19 nucleotide deletion disrupting the poison exon 3′ splice site was sufficient to completely exclude the poison exon in fibroblast mRNA. In contrast, poison exon inclusion was not impacted by upstream intronic deletion (Fig 2D). This result suggests a limited deletion of necessary splicing sequences surrounding the *Smndc1* poison exon is sufficient to prevent inclusion of the poison exon in our mouse model. As both the poison exon and 3′ splice site deletion alleles effectively prevented exon inclusion and are therefore functionally equivalent, we aggregated the results of both the poison exon deletion model as well as the 3′ splice site deletion model in subsequent analyses.

### Generation of an *A*. *thaliana* model lacking the *SPF30* poison exon

We next sought to model the corresponding loss of the *SPF30* poison exon in *A*. *thaliana*. The *SPF30* locus is composed of eight exons, with four annotated splice variants. To generate CRISPR/Cas9-edited *A*. *thaliana* lines, we used floral dip transformations to transfer nuclear-localized Cas9 protein under egg cell-specific promotion and gRNA sequences under constitutive promotion. pgRNAs were selected for optimal off-target scores and were designed to flank the poison exon 3′ splice site, as well as the upstream intron as a control for the effects of regional editing (S2 Table and Fig 2E). We confirmed deletion alleles present in T1 plants through PCR and Sanger sequencing at the appropriate DNA locus, and selected a poison exon deletion allele that removed the 3′ splice site as well as 48 nucleotides of the *SPF30* poison exon (S4C Fig).

To ensure the deletion at the *SPF30* poison exon indeed prevented poison exon inclusion and did not introduce aberrant splice isoforms, we collected and pooled T2 seedlings, treated with CHX to inhibit NMD, and performed RT-PCR. For efficiency and to gather sufficient amounts of RNA, RT-PCR was performed using a mixed pool of both heterozygous and homozygous T2 seedlings. Inclusion of the poison exon was reduced by 80.65% in the *SPF30* poison exon deletion plants relative to the intronic control plants (Fig 2F). Importantly, no novel alternative splice isoforms were introduced through deletion of the *SPF30* poison exon. Seeds from additional heterozygous T1 plants were planted, resulting plants were genotyped, and confirmed homozygous plants were selected for further phenotyping.

### The *Smndc1* poison exon regulates transcript and protein abundance *in vivo*

**Fig 3:**
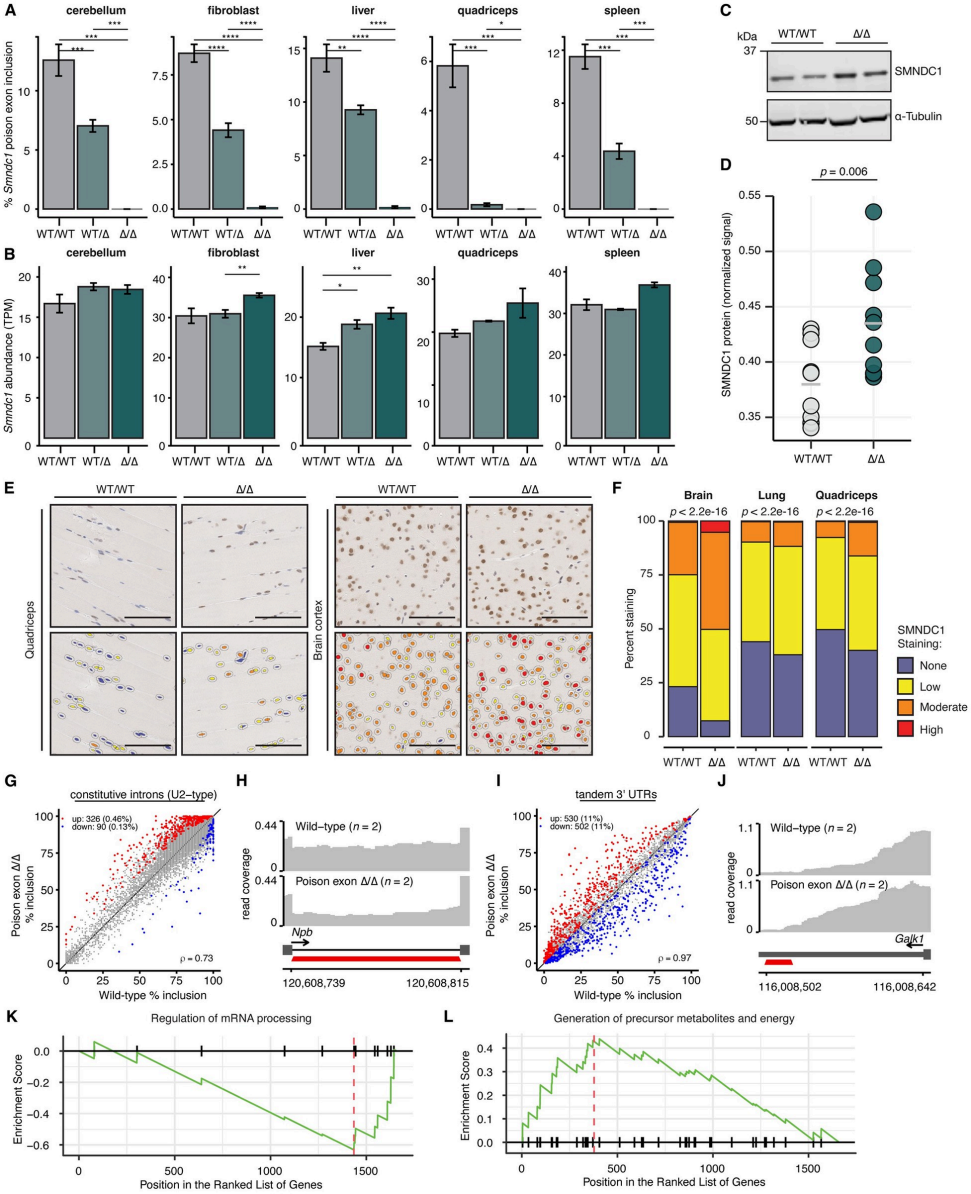
Loss of *Smndc1* poison exon alters global transcript and protein abundance, while steady state RNA splicing is modestly affected. **A**, Quantification of RNA-sequencing (RNA-seq) reads supporting *Smndc1* poison exon (PE) inclusion. RNA was collected from mice which were wild-type (WT/WT), heterozygous (WT/Δ), or homozygous (Δ/ Δ) for the *Smndc1* PE. P-values computed using two-sided Wilcoxon rank-sum test (*p < 0.05, **p < 0.01, ***p < 0.001, ****p < 0.0001). *n* = 2 biological replicates per genotype (cerebellum, spleen, quad), *n* = 6 biological replicates per genotype (liver, fibroblast). **B**, as in (**A**) demonstrating total *Smndc1* mRNA transcript abundance (TPM). **C**, Representative Western blot of SMNDC1 signal from wild-type (WT/WT) and PE null (Δ/Δ) mouse liver lysate. *n* = 2 biological replicates per genotype. **D**, Quantification of SMNDC1 signal from Western blots generated with wild-type (WT/WT) and PE null (Δ/Δ) mouse liver lysate. Signal normalized to alpha-tubulin. P-value computed using one-sided Student’s T-test. *n* = 10 animals per genotype. **E**, (Left) Representative immunohistochemistry images of quadriceps and brain cortex collected from wild-type and *Smndc1* PE null littermates, stained with diaminobenzidine (DAB) for SMNDC1. (Right) The same representative images with SMNDC1 nuclear staining classified using HALO software as negative (blue), weak (yellow), moderate (orange), or strong (red). Scale bar: 100 μm. **F**, Stacked barchart of SMNDC1 staining intensities from tissues using quantification method in (**D**). P-values computed using multinomial proportion test. *n* = 3 mice (PE Δ/Δ) and *n* = 2 mice (WT/WT). **G**, Scatter plots of constitutive intron splicing from cerebellum RNA-seq. Comparison between wild-type and *Smndc1* PE Δ/Δ mice. Red and blue points represent increased and decreased spliced isoforms in the poison exon null samples, respectively. *n* = 2 biological replicates per genotype. **H**, Representative RNA-seq coverage plot of intronic splicing of *Npb* from cerebellum samples. *n* = 2 biological replicates per genotype. **I**, as in (**G**), demonstrating tandem 3′ UTR reads from cerebellum RNA-seq. **J**, as in (**H**), demonstrating a tandem 3′ UTR event in *Galk1*. **K**, Gene set enrichment analysis (GSEA) of differentially spliced events in cerebellum from *Smndc1* Δ/Δ samples relative to WT samples (NES = -1.90, Benjamini-Hochberg adjusted *p* = 0.004). **L**, GSEA of differentially spliced events in cerebellum from *Smndc1* Δ/Δ samples relative to WT samples (NES = 1.56, Benjamini-Hochberg adjusted *p* = 0.012).

Having established murine *Smndc1* poison exon knockout models, we next sought to determine how loss of the poison exon impacted global *Smndc1* mRNA and protein abundance at the organism scale. We began by gathering tissues, including primary fibroblasts, for RNA-seq from three types of mice: poison exon null mice, heterozygous mice, and wild-type littermates. Consistent with RT-PCR data, poison exon null mice demonstrated few to no junction reads aligning to the *Smndc1* poison exon (Fig 3A). Meanwhile, heterozygous mice demonstrated intermediate poison exon-aligning reads, consistent with reduced allelic dose. Loss of the poison exon further correlated with increases (between 10.50–36.15%) in total *Smndc1* mRNA transcript abundance across all tissues and cell lines assayed (Figs 3B and S5A and S3 Table).

To test whether an increased abundance of *Smndc1* mRNA transcript corresponds with greater protein abundance, we quantified SMNDC1 protein with immunoblotting. SMNDC1 mean abundance was significantly higher in both liver samples (14.50%; *p* = 0.012) and cerebellum samples (8.97%; *p* = 0.05) from poison exon null mice relative to wild-type mice, consistent with de-repression of *Smndc1* mRNA transcript (Figs 3C, 3D and S5B–S5D). Similarly, SMNDC1 immunohistochemistry staining demonstrated increased nuclear protein abundance in poison exon null tissues across multiple tissues assayed (Fig 3E). Using a HALO software image classifier, we quantified cells from each tissue with no, low, moderate, or high SMNDC1 nuclear staining signal. In tested brain, lung, and quadriceps tissue, we detected significant genotype-dependent increases (*p* < 2.2 x 10^−16^) in the proportions of cells staining for SMNDC1 in all three tissues (Fig 3F and S1 Appendix). Together, these results demonstrate that loss of the *Smndc1* poison exon impacts global mRNA and protein abundance in steady-state cells and tissues. Specifically, the greatest fold-change in both overall SMNDC1 protein staining (1.26-fold increase) and “strong” SMNDC1 staining (9.34-fold increase) was in brain samples.

Given that deletion of the *Smndc1* poison exon leads to increased SMNDC1 protein abundance and because SMNDC1 is known to interact with many mRNA splicing and processing proteins [31], we next sought to determine if global splicing was impacted by poison exon loss. We focused on RNA-seq data from mouse cerebellar transcriptome, given the high levels of IHC staining in brain samples and 8.97% increase in cerebellum protein signal via Western blot in the setting of *Smndc1* poison exon loss (S5D Fig). Loss of the *Smndc1* poison exon contributed to increased splicing of hundreds of intronic events compared to wild-type transcriptomes, such as increased intronic splicing of *Npb* (Fig 3G and 3H and S4 Table). Increased intronic splicing is consistent with greater abundance of SMNDC1 protein, given its importance in spliceosome assembly, and also consistent with our previous findings in *SMNDC1* poison exon null neoplastic cell lines [8]. In addition to intron splicing, loss of the *Smndc1* poison exon impacted differential polyadenylation and the 3′ UTR of many transcripts within the cerebellum (Fig 3I and S4 Table). Loss of the *Smndc1* poison exon was associated with distal polyadenylation site usage in 11% of queried transcripts, while a similar percentage of transcripts shifted usage toward a more proximal polyadenylation site, such as the 3′ UTR of *Galk1* (Fig 3I and 3J). In addition to cerebellum transcripts, hundreds of differential splicing events were identified between wild-type and poison exon null samples sourced from both liver and primary fibroblast cell lines (S5E and S5F Fig and S5 and S6 Tables).

To determine whether *Smndc1* poison exon deletion preferentially impacted splicing within particular functional pathways, we performed gene set enrichment analysis (GSEA) [32,33]. GSEA revealed that splicing events differentially perturbed in *Smndc1* poison exon null samples were most associated with the regulation of mRNA processing (*p* = 0.004, Fig 3K) and also associated with the generation of precursor metabolites and energy (*p* = 0.012, Fig 3L). To further compare transcriptomic changes across multiple tissues, we performed Gene Ontology (GO) analysis using MSigDB Hallmark gene sets [34]. SMNDC1 perturbation resulted in differentially spliced transcripts which were enriched in the hallmark pathways “G2M checkpoint,” “MTORC1 signaling,” and “Oxidative Phosphorylation” across liver, cerebellum, and fibroblast samples (S6A Fig). Many shared pathways exist between tissues, including multiple metabolic pathways (adipogenesis, glycolysis, oxidative phosphorylation).

In addition to differentially spliced functional pathways, we also examined differentially expressed pathways. Many pathways related to RNA biological processing were enriched in *Smndc1* poison exon null samples, including ncRNA and rRNA metabolic processing, RNA processing, and RNA metabolic processing (S6B Fig). Additionally, multiple splicing factors were differentially expressed in *Smndc1* poison exon null samples, including *Scaf8*, *Srek1*, *Phrf1*, and *Celf1* (S6C Fig). These findings indicate that in *Smndc1* poison exon null mice, RNA processing is disrupted due to alterations in splicing and expression, along with signs of metabolic disruption at the mRNA level.

### Increased SMNDC1 protein levels in *SMNDC1* poison exon-null cells are responsible for increased intron excision

To assess whether the global transcriptomic changes that we observed following *Smndc1* poison exon deletion were caused by the increased SMNDC1 protein abundance, we partially knocked down *SMNDC1* using RNA interference in the context of *SMNDC1* poison exon loss and performed RNA-seq. We used a previously established and validated human cell line expressing inducible Cas9 with stably integrated pgRNAs targeting a control region (*AAVS1*) or the *SMNDC1* poison exon [8], as these cells are very amenable to experimental manipulation. We previously demonstrated that this poison exon knockout cell line exhibited increased constitutive intron splicing [8], concordant with our observations in *Smndc1* poison exon KO mouse cells in the current study, making it well-suited to this rescue experiment.

We first validated that CRISPR-mediated targeting of the *SMNDC1* poison exon in these cells (pg*SMNDC1*-PE) results in increased SMNDC1 at the protein level (S7A Fig). Next, we used two unique siRNAs to knock down coding *SMNDC1* transcripts in the pg*SMNDC1*-PE cells, performed RNA-seq following 48 hours of siRNA knockdown, and measured levels of SMNDC1 mRNA and protein. These experiments confirmed the expected reduction in *SMNDC1* transcript abundance for both siRNAs as well as restoration of SMNDC1 protein levels to near-baseline levels (S7A and S7B Fig).

We then tested how restoration of near-baseline levels of SMNDC1 protein levels affected splicing in the context of *SMNDC1* poison exon loss. CRISPR-mediated targeting of the SMNDC1 poison exon (pg*SMNDC1*-PE) resulted in modestly increased splicing of constitutive introns, as expected based on our prior publication [8] and mouse results here, along with balanced alterations in cassette exon inclusion (S7C and S7D Fig and S7 Table). In contrast, treating these cells with either unique siRNA to knock down *SMNDC1* resulted in globally less efficient splicing of constitutive introns and preferential exclusion of hundreds of cassette exons (S7E–S7H Fig and S7 Table). Together, these data indicate that maintaining SMNDC1 protein level homeostasis is important for global splicing and that disruption of normal SMNDC1 protein abundance via poison exon deletion leads to global splicing dysregulation.

### Loss of the *Smndc1* poison exon and the *SFP30* poison exon results in reduced size in mouse and *A*. *thaliana* models

We next investigated the developmental and morphological phenotypes associated with alteration of *Smndc1* in our mouse models. *Smndc1* is considered an essential gene *in vitro* [22], so to serve as a positive control we generated a *Smndc1* gene knockout mouse model through pgRNA-directed CRISPR/Cas9 editing (S8A Fig). We used pgRNA to disrupt coding exon 4, resulting in a founder mouse with a 5-bp frameshifting deletion allele which was backcrossed (S8A and S8B Fig). Resulting backcrossed, heterozygous mice expressed less SMNDC1, indicating that the deletion allele may have impeded the translation process of *Smndc1* mRNA transcripts into functional proteins (S8C Fig). Furthermore, no homozygous *Smndc1* knockout mice were born (*n* = 52), which is a statistically significant deviation from normal Mendelian inheritance (*p* = 7.93e-5; S8D Fig). These data suggest *Smndc1* gene knockout is not developmentally viable.

**Fig 4:**
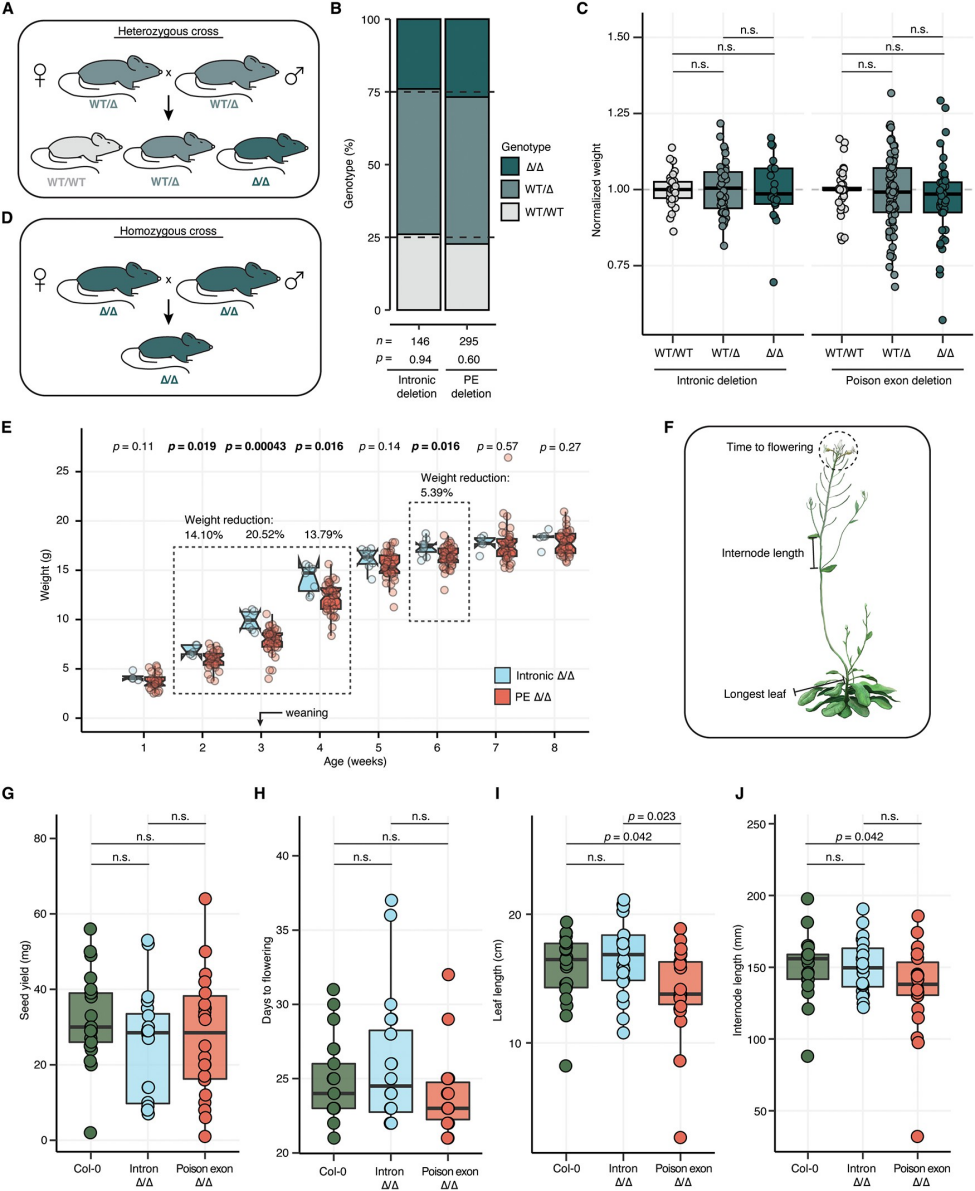
Reduced size characterizes the *Smndc1* and *SPF30* poison exon null organisms. **A**, Schematic of heterozygous mouse crosses that generated the data in panels (**B**) and (**C**). Possible pup genotypes are wild-type (WT/WT), heterozygous (WT/Δ), or homozygous (Δ/Δ) for *Smndc1* poison exon (PE). **B**, Genotype percentages of pups born to heterozygous crosses of intronic deletion and *Smndc1* PE deletion lines. P-values generated through Chi-squared testing. **C**, Quantification of pup weight at weaning from heterozygous crosses of either the intronic control line (*n* = 93 mice) or *Smndc1* poison exon deletion lines (*n* = 172 mice). Weight normalized to the median weight of all wild-type pups in each respective litter. Statistical significance was assessed by the Wilcoxon-rank sum test. **D**, Schematic of homozygous mouse crosses generating the data in panel (**E**). **E**, Quantification of homozygous female pup weight at postnatal weeks 1–8. Weight measured for all pups born to homozygous breeder pairs from both the *Smndc1* intronic deletion and *Smndc1* PE deletion lines. Statistical significance was assessed by the Wilcoxon-rank sum test (*n* = 62 mice). **F**, Schematic demonstrating phenotypes measured in *A*. *thaliana* genetically modified plants. Modified image from DataBase Center for Life Science, licensed under CC BY 4.0 (https://doi.org/10.7875/togopic.2021.057). **G-J**, Phenotypic assessment of *wild-type* Col-0, intronic deletion, and the *SPF30* PE deletion lines. *n* = 20 biological replicates per line. Statistical significance was assessed by the Wilcoxon-rank sum test. In (**G**), quantification of total seed mass yielded per plant in milligrams. In (**H**), measurement of days to flowering (onset of flowering, 1 centimeter (cm) inflorescence). In (**I**), quantification of maximal plant leaf length in cm at onset of flowering. In (**J**), quantification of internode length in millimeters.

We next aimed to determine if there was embryonic lethality associated with alteration of non-coding elements of *Smndc1*, specifically in the poison exon deletion model. To test this, we established heterozygous crosses and genotyped all offspring derived from these crosses at weaning (Fig 4A). Genotyped offspring from both the poison exon deletion and control lines adhered to expected Mendelian ratios, suggesting that loss of the *Smndc1* poison exon does not impair viability under heterozygous breeding conditions (Fig 4B). We also found no significant difference in litter sizes or sex ratios of pups born to either the Smndc1 poison exon or control deletion lines (S9A and S9B Fig). This demonstrates that the reproductive success of poison exon heterozygous mice is comparable to that of the control counterparts.

To assess gross morphology of the *Smndc1* poison exon null mice generated from heterozygous crosses, we weighed mice at weaning and over time. At weaning (21 days old), the *Smndc1* poison exon null mice were equivalently sized to wild-type and heterozygous littermates and did not demonstrate gross morphological differences (Fig 4C). Poison exon null mice exhibit a trend towards statistically significant reduced birth rate and reduced size at weaning compared to littermates, but these trends do not reach significance (Fig 4B and 4C). Aging poison exon null mice up to 20 months demonstrated no change in overall weight nor mortality rate when compared to wild-type and heterozygous littermates (S9C–S9E Fig). Additionally, we performed complete gross necropsy on male poison exon null, heterozygous, and wild-type littermates (2 replicates per genotype). The following tissues were evaluated by a board-certified staff pathologist: liver, spleen, kidney, heart, gonad, and brain. While there were no genotype-dependent changes in weight or relative organ size, loss of the poison exon was associated with proliferative and necrotizing polyarteritis in one poison exon null mouse (S10A–S10E Fig). This could not be differentiated from idiopathic systemic inflammation, and further replicates are needed to assess the significance of this finding.

A key component of organism fitness is the ability to reproduce and generate viable offspring. Therefore, we next evaluated the reproductive success of the *Smndc1* poison exon null mice by establishing poison exon null breeder pairs (Fig 4D). These breeder pairs were able to reproduce at normal frequency and with typical litter sizes. We assessed the weight of homozygous offspring of poison exon null crosses every week from one week after birth to eight weeks of age as a proxy for offspring health. Compared to homozygous control offspring, both male and female *Smndc1* poison exon null mice were significantly smaller during early postnatal development. In female *Smndc1* poison exon null offspring, we observed 14.10% weight reduction (*p* = 0.019), 20.52% weight reduction (*p* = 0.00043), and 13.79% weight reduction (*p* = 0.016) at postnatal weeks 2, 3, and 4, respectively (Fig 4E). The weight reduction persisted until postnatal week 6 (5.39% weight reduction, p = 0.016, Fig 4E). Similarly, male *Smndc1* poison exon null mice demonstrated 13.01% weight reduction (*p* = 0.014) and 10.68% weight reduction (*p* = 0.03) at postnatal weeks 3 and 4, respectively (S11A Fig). These phenotypes were notable due to striking weight changes, temporal specificity, and presence solely for homozygous offspring born to homozygous—and not heterozygous—breeders.

We next tested whether the *A*. *thaliana* model exhibited gross morphological phenotypes or other signs of reduced fitness corresponding to the loss of the *SPF30* poison exon. We chose to examine commonly measured life history traits of *A*. *thaliana*, including longest leaf length at the onset of flowering, internode length on the primary inflorescence, time to flowering in days, and total seed yield (Fig 4F). Total seed yield and days to flowering were not impacted by loss of the poison exon (Fig 4G and 4H). In contrast, *A*. *thaliana* homozygous for the *SPF30* poison exon demonstrated a significant reduction in leaf length and internode length relative to intronic deletion plants and Col-0 plants (Fig 4I and 4J). We conclude that loss of the *SPF30* poison exon in *A*. *thaliana* results in growth restriction without impact to reproductive phenotypes.

## Discussion

Regulatory mechanisms like unproductive splicing play a critical role in controlling the abundance of RNA-binding proteins. Yet, most highly conserved splice isoforms in RNA-binding proteins have not been functionally examined. To specifically modulate isoform splicing *in vivo*, we applied CRISPR/Cas9 pgRNA targeting to generate specific and minimally disruptive gDNA edits. Using this approach, this study is the first to directly knock out poison exons in multiple model organisms. This technique offers the potential for exploring the functional significance of other poison exons in RNA-binding proteins.

We demonstrate that loss of the *Smndc1* poison exon and consequential increase in SMNDC1 protein levels result in dysregulated splicing and expression of hundreds of transcripts, preferentially affecting genes involved in RNA processing and metabolism, consistent with the recent report of SMNDC1’s role in regulating pancreatic hormone expression [35]. Alteration to these pathways could lead to neurological, physiological, or behavioral changes that may explain the reduced weight observed in *Smndc1* poison exon null mice reared by *Smndc1* poison exon null breeders. It is intriguing that female pups demonstrated greater weight reduction and for greater duration than male pups. It is possible that the downstream splicing changes resulting from the dysregulation of *Smndc1* in our models are more likely to be phenotypically disruptive in female mice, potentially due to differences in hormone regulation and neurocognitive development, which is sex-specific in mice and humans [36–38].

Deeper characterization of specific downstream molecular mechanisms is needed to fully understand the phenotype demonstrated in the *Smndc1* poison exon null mice. For example, while we performed RNA-seq in mice ranging from 5–7 weeks old, we have not examined the transcriptomes of mice during early development, which could be critical to understanding the molecular mechanisms driving phenotypes displayed in our models. The observed temporal specificity, with reduced weight around weaning age in *Smndc1* poison exon null pups, suggests there might be an underlying parental phenotype in *Smndc1* poison exon null mice. Close examination of parental behavior, including milk production characteristics and weaning behaviors, could enlighten the specific morphological phenotypes of mice reared by *Smndc1* poison exon null breeders but not heterozygous breeders.

Many splicing factors contain poison exons that are either highly conserved or overlap with ultraconserved DNA elements (UCEs) [13,14]. Of the 481 UCEs present in the human genome, the majority are found within non-coding genomic regions, such as poison exons or enhancers [39,40]. Numerous deletion studies have targeted ultraconserved enhancers *in vivo*, but only a few have reported developmental or tissue-specific phenotypes associated with homozygous or hemizygous mice [41–44]. Among these deletion models, there are no obvious changes to overall viability or reproduction, a phenomenon recapitulated in our model of deletion of a highly conserved poison exon. Nonetheless, it is important to note that the current study and prior studies of UCE deletion do not recapitulate native environmental selective pressures. It is possible and perhaps even probable that outside of the highly controlled laboratory setting or across multiple generations, the phenotypic consequences of loss or mutation of poison exons and other highly conserved genomic elements would be much greater.

## Materials and methods

### Ethics statement

Animal husbandry was conducted in accordance with the Guidelines for the Care and Use of Laboratory Animals and was approved by the Institutional Animal Care and Use Committees at Fred Hutchinson Cancer Center.

### Identification of orthologs and sequence alignment

To identify protein orthologs of SMNDC1, we employed Basic Local Alignment Search Tool (BLAST) and reciprocal best hit (RBH) analysis. The nucleotide sequence alignment was generated with the Clustal Omega online tool [45]. We performed a BLASTP (protein-protein) search using human SMNDC1 and *A*. *thaliana* SPF30 sequences as queries against the protein database of each target species. The BLASTP search was conducted using the NCBI BLAST+ suite (version 2.14.0+) with default parameters and using the BLOSUM62 substitution matrix. The top hits from the initial BLASTP search were subjected to a reciprocal BLASTP search against the protein database of the query species. Exon DNA sequences were aligned using EMBOSS Needle pairwise sequence alignment with default settings.

### Mouse model generation and genotyping

All deletion alleles were generated by CRISPR/Cas9-mediated genome editing with paired guide RNAs (pgRNAs), as previously described [8,46]. High-quality pgRNA seed sequences (S2 Table) were selected using the Benchling guide design tool, confirmed by GuideScan, and analyzed for specificity using CasOffinder. pgRNAs were produced through cloning-free *in vitro* transcription as previously described [47] using the MEGAshortscript T7 Transcription Kit (Life Technologies). Products were purified using the MEGAclear Transcription Clean-up Kit (Life Technologies). Cas9 mRNA (50 ng/μL) and pgRNA (25 ng/μL) were microinjected into single-cell zygotes derived from B6(Cg)-Tyrc-2J/J inbred mice (JAX strain #000664). Microinjected embryos were transplanted into pseudopregnant CD-1 recipients.

Founder animals were genotyped using ear punch tissue with primers that flanked the expected deletion loci by 100 to 200 bases (S8 Table). The resulting amplicons were further characterized by Sanger sequencing. We selected multiple heterozygous founder animals from each deletion line for germline transmission confirmation breeding, to ensure the identified deletion alleles would be transmitted to the next generation. Once allele transmission was confirmed, we chose one allele for each deletion type (S4B Fig) to continue backcrossing with C57BL/6 breeders (JAX strain #000664) for at least four generations. Each backcrossed generation was genotyped to ensure transmission of the correct deletion allele.

To genotype founder mice and subsequent generations, ear tissue was collected when the mice were two to three weeks old and digested in Lysis M Buffer (Macherey-Nagel) and Proteinase K (Qiagen) for five minutes at room temperature. This was followed by standard PCR using appropriate primers and gel electrophoresis (S8 Table). PCR products were subsequently Sanger sequenced when alleles were indistinguishable through gel electrophoresis (S8 Table). After the establishment of backcrossed lines, genotyping was performed using quantitative genomic PCR (Transnetyx, Cordova, TN). For all downstream molecular studies with backcrossed mice, mouse age was controlled between 4–6 weeks old (S9 Table).

### *A*. *thaliana* model generation

Seeds from CRISPR/Cas9-generated T1 plants were provided by Creative Biogene (Shirley, NY, USA), after careful selection of gRNA with optimal off-target scores (S2 Table). Due to local sequence constraints, only one gRNA was used to target the upstream intronic region. Genetically modified plants contain targeting gRNA sequences driven by the Arabidopsis U6-26 promoter, an EC1.1-promoted Cas9 protein with a nuclear localization signal, and a bar gene selectable marker (basta resistance gene). To genotype plants, we collected genomic DNA from leaf samples using the cetyltrimethyl ammonium bromide (CTAB) method, performed standard PCR with appropriate primers which flanked the expected deletion locus by 100–200 bp (S8 Table), and followed this with gel electrophoresis and Sanger sequencing. Seeds collected from heterozygous plants were planted, and the resulting progeny were screened to identify homozygous plants, from which homozygous seeds were collected.

### Isolation of mouse fibroblasts

Isolation of primary fibroblasts was performed from mouse ear and tail tissue as previously outlined [48], with the following modifications. We euthanized eight-week-old wild-type, heterozygous, and homozygous littermates, collected ear and tail tissues, and immediately carried out digestion in a solution of 15 U/mL collagenase and 0.13% dispase. Following digestion and filtration, the cells were cultured in Ham’s F10 medium with 10% FBS, 1% penicillin, and 1% streptomycin. Once the fibroblasts reached confluency, they were dissociated and split at a ratio of 1:5.

### RT-PCR

To detect poison exon-containing RNA isoforms in cell lines and *A*. *thaliana* seedlings, we used cycloheximide (CHX) to inhibit NMD prior to RNA extraction. Cell lines were treated with media containing 100 μg ml−1 CHX for three hours before collection in TRIzol reagent (Invitrogen). *A*. *thaliana* seedlings (24 days) were removed from the soil, rinsed in buffer (0.046 g/L Murashige and Skoog Plant Salt Mixture, 0.3 g/L sucrose, pH 5.8), and vacuum-infiltrated with 10 μg ml−1 CHX in buffer for 10 minutes, as previously described [10]. After vacuum infiltration, seedlings were incubated in the CHX buffer for three additional hours at room temperature prior to flash-freezing and immediately homogenization in TRIzol reagent (Invitrogen).

Total RNA was extracted using the Direct-zol RNA MiniPrep (Zymo Research). cDNA was synthesized using SuperScript IV Reverse Transcriptase (Thermo Scientific) according to the manufacturer’s protocol. RT–PCR was performed with Phire Green Hot Start II DNA polymerase (Thermo Scientific) and gene-specific primers (S8 Table). Amplicons were analyzed and quantified using agarose gel electrophoresis. Band intensity was then quantified using FIJI/ImageJ. Supporting raw data for RT-PCR band quantification is provided (S10 Table).

### RNAi-mediated knockdown experiment

HeLa iCas9 cells with stably transduced pgRNAs targeting *AAVS1* or the *SMNDC1* poison exon were grown in media containing 1 μg/mL doxycycline for one week to induce Cas9 expression. HeLa cells were then seeded into 6-well plates (200,000 cells/well) immediately prior to transfection. For RNA interference, siRNA sequences were GAAUUGCUGAUAAACCUAUTT (siRNA1, Silencer Select s20101, Thermo Fisher Scientific), GAGUGUGACUGGUAAAGUUTT (siRNA2, Silencer Select s20102, Thermo Fisher Scientific), or Silencer Select Negative Control No. 1 (4390843, Thermo Fisher Scientific). Either 2.4 μL or 4.8 μL of siRNA was added to 150 μL OptiMEM, then added to 150 μL OptiMEM with RNAiMAX (Thermo Fisher Scientific), and incubated for 10 minutes at room temperature. The transfection mix was added drop-wise to seeded cells for a final concentration of 10 μM or 20 μM. Cells were collected after 48 hours.

### Mouse aging and necropsy

Mice were kept under standard housing conditions. To evaluate the impact of aging, we tracked their body weight monthly. The mice were gently restrained and weighed on a digital scale with a precision of 0.01 grams. This weight data was utilized to monitor age-related changes and assess general health throughout the study period, and supporting raw data is provided (S11 Table).

For necropsy, 6-month-old male mice were euthanized with CO_2_ to retrieve organs (6 replicates total). Organs were washed with deionized water before fixation in 4% paraformaldehyde. The tissues were processed routinely, and sections were stained with hematoxylin and eosin. The specimens were interpreted by a board-certified staff pathologist, in a blinded fashion.

### Immunohistochemistry and HALO analysis

Tissue embedding and staining was performed by the Experimental Histopathology Core at Fred Hutchinson Cancer Center. Mouse SMNDC1 was detected using a mouse polyclonal antibody (Thermo Fisher PA5-31148) at 1:8000 dilution following optimization by the Experimental Histopathology core facility. Staining was performed using a BOND RX autostainer (Leica Biosystems). Imaging was performed using an Aperio ImageScope (Leica Biosystems), and image analysis was completed using HALO Image Analysis software.

### Western blotting

Total protein lysates were prepared in RIPA buffer (Cell Signaling) with 1mM Pefabloc (Sigma-Aldrich) and quantified using the Pierce 660 nm Protein Assay Reagent (Thermo Fisher). Protein lysates were electrophoretically separated and transferred to nitrocellulose membrane using NuPAGE systems (Thermo Fisher). Membranes were blocked with Odyssey Blocking Buffer (LI-COR Biosciences) for one hour at room temperature. Primary antibody incubation was performed overnight at 4°C in Intercept Antibody Diluent (LI-COR Biosciences), with SMNDC1 (Thermo Fisher PA5-31148, 1:1000) and alpha-tubulin (Sigma-Aldrich T6199, 1:1000) primary antibodies. IRDye (LI-COR Biosciences) secondary antibodies were used for detection and imaging using the Odyssey CLx Imager (LI-COR Biosciences). Protein blot analysis and quantification were performed using FIJI/ImageJ and quantification is provided (S12 Table).

### Protein overexpression in B16-F10 cells

Mouse *Smndc1* coding sequence was expressed in the PCMV6-Kan/Neo backbone (OriGene). To generate a construct containing *A*. *thaliana SPF30* coding sequence, mouse *Smndc1* cDNA sequence was dropped out with EcoRI and MluI digestion and *A*. *thaliana* cDNA fragment was inserted using Gibson assembly. The constructs were transiently transfected in serum-free media with Lipofectamine 3000, using 0, 0.25, 1, or 2.5 μg per 400,000 B16-F10 cells in a 6 well plate. RNA was collected for RT-PCR 24 hr post-transfection.

### RNA-seq library preparation and data analysis

Fibroblast RNA was extracted using Direct-zol RNA MiniPrep kit (Zymo Research), according to the manufacturer’s instructions. For tissue RNA extractions, tissue was homogenized in TRIzol reagent with zirconium beads prior to RNA extraction, using the Zymo Direct-zol RNA Miniprep kit. A minimum of 500 ng of high-quality RNA was used as input for library preparation. Poly(A)-selected unstranded libraries were prepared using a TruSeq protocol and sequenced using an Illumina sequencer to obtain 100 bp paired-end reads.

RNA-seq reads were processed as previously described [49]. In brief, FASTQ files were trimmed to remove sequencing adapters and then aligned to the hg19/GRCh37 reference assembly. This assembly was created by merging the UCSC knownGene gene annotation [50], Ensembl gene annotation [51], and MISO isoform annotation [52]. Read alignment and expression estimates were generated with RSEM (RNA-seq by expectation maximization) [53], Bowtie [54], and TopHat [55], and isoform ratios were quantified with MISO v.2.0 [52]. Aligned reads from mapping steps were merged to generate BAM coverage plots. Significantly differentially spliced events met the following criteria: at least 20 identifying reads in each sample, a minimum Bayes factor of 5, and a minimum of 10% change (absolute scale) in isoform ratio or minimum fold-change of 2 (log2 scale) in absolute isoform ratio.

As the mouse *Smndc1* poison exon was not annotated in the described RNA-seq mapping method, RNA-seq reads were manually mapped to the *Smndc1* poison exon inclusion and exclusion isoforms using Bowtie. No mismatched nucleotides were allowed, junction-spanning reads were filtered to require a minimum overhang of 6 nucleotides, and resulting counts were normalized to the length of the mapping index.

All analyses and visualization were performed in the R programming environment, with tools from Bioconductor and dplyr, ggplot2, tidyverse packages.

### Plant growth and phenotyping

For all experiments, control and edited plant lines were grown under long day conditions (16 hour light/8 hour dark), with fluorescent bulbs at 70–80 μmol m−2 s−1. The room was maintained at 22°C and 50% relative humidity. The wild-type Col-0 seeds were collected, stored, and planted at the same time as the seeds of the genetically modified lines. All genotypes were grown in a random block design and plant trays were regularly rotated to account for location-dependent growth effects. Longest leaves were measured as the longest leaf at time of bolting (onset of flowering, defined by the presence of a 1 cm primary inflorescence). Internode length was measured from the primary inflorescence. Seed yield was measured as the total mass of all seeds collected from a fully dried plant. Supporting data is provided (S13 Table).

## Supporting information

S1 Table Reciprocal best hit analysis across species. For each species queried, includes E-value, percent identity, protein identifier, and result of reciprocal BLASTP.
(XLSX)

S2 Table Sequences of pgRNAs used in model generation. Summary of pgRNA sequence for *M. musculus* and *A. thaliana*. (XLSX)

S3 Table *Smndc1* expression across mouse models and tissue samples. Summary of *Smndc1* expression from RNA-seq data, including mouse identifiers, origin tissue, and gene expression (TPM).
(XLSX)

S4 Table Differentially spliced events in wild-type and *Smndc1* poison exon null cerebellum samples. Summary of differential splicing events between samples, including event annotation, genomic coordinates, gene identifier, NMD relevance, PSI value, log fold change, and Bayes factor.
(XLSX)

S5 Table Differentially spliced events in wild-type and *Smndc1* poison exon null liver samples. Summary of differential splicing events between samples, including event annotation, genomic coordinates, gene identifier, NMD relevance, PSI value, log fold change, and Bayes factor.
(XLSX)

S6 Table Differentially spliced events in wild-type and *Smndc1* poison exon null fibroblast samples. Summary of differential splicing events between samples, including event annotation, genomic coordinates, gene identifier, NMD relevance, PSI value, log fold change, and Bayes factor.
(XLSX)

S7 Table Differentially spliced events in HeLa-iCas9 cells treated with siRNA. Summary of differential splicing events between samples, including event annotation, genomic coordinates, gene identifier, NMD relevance, PSI value, log fold change, and Bayes factor.
(XLSX)

S8 Table Primers used for Sanger sequencing, gDNA PCR, and RT-PCR. Oligo sequences, applications, and identifiers.
(XLSX)

S9 Table Identification of mice used in molecular experiments. Includes strain, sex, genotype, and application.
(XLSX)

S10 Table RT-PCR quantification supporting data. (XLSX)

S11 Table Mouse weight and aging supporting data. (XLSX)

S12 Table Western blot quantification supporting data. (XLSX)

S13 Table *A*. *thaliana* phenotyping supporting data. Summary of plant location including plot, row, column; plant phenotype including leaf length, days until flowering, length of first internode, seed yield.
(XLSX)

S1 Fig Conservation of nucleotide sequence and NMD-targeting in the human *SMNDC1*, mouse *Smndc1*, and *A*. *thaliana SPF30* poison exons. **A**, Schematic representation of the intron-exon structure of *SMNDC1*, *Smndc1*, and *SPF30*, respectively. Introns and exon lengths are scaled appropriately to each gene, but genes are not scaled to each other. Highlighted are upstream coding exons (blue), poison exons (red), and downstream coding exons (green). **B**, Alignment of human, mouse, and *A*. *thaliana* DNA sequences from coding and poison exons of *SMNDC1*, *Smndc1* and *SPF30*, respectively. Highlighted are upstream coding exon (blue), poison exon (red), and downstream coding exon (green). **C**, BAM coverage plot of *SPF30* gene structure and mapped read coverage in samples treated with or without cycloheximide (CHX). Poison exon supporting reads highlighted in yellow. The poison exon (green box) was previously unannotated.
(TIF)

S2 Fig Inspection of NMD-targeted transcripts from genes encoding core spliceosome factors in *A*. *thaliana*. **A-E**, BAM coverage plots depicting transcript structure (blue) and mapped read coverage (gray) from samples treated with or without cycloheximide (+ CHX and–CHX, respectively). Poison exon supporting reads are highlighted in yellow. **A**, AT2G02570 (*A*. *thaliana*), an ortholog to *SMNDC1* (human); **B**, AT3G50670 (*A*. *thaliana*), an ortholog to *SNRP70* (human); **C**, AT1G03140 (*A*. *thaliana*), an ortholog to PRPF18 (human); **D**, AT5G52545 (A. thaliana), an ortholog to *RBM18* (human); **E**, AT1G07350 (*A*. *thaliana*), an ortholog to *TRA2A* (human).
(TIF)

S3 Fig Transient overexpression of SMNDC1 in mouse melanocytes. **A**, Western blot demonstrating SMNDC1 signal from mouse melanocytes using a transient overexpression system with increasing amounts of SMNDC1 expression. SMNDC1 signal is normalized to alpha-tubulin signal.
(TIF)

S4 Fig Targeting and validation of *Smndc1* and *SPF30* deletion alleles. **A**, Paired guide RNAs (pgRNAs, colored boxes) designed to disrupt the *Smndc1* poison exon (red box). pgRNAs target regions of high placental conservation or the upstream intronic region. **B**, Sanger sequencing of mouse lines with 254 base pair (bp) deletion of the *Smndc1* poison exon (PE), 19 bp deletion of the *Smndc1* PE 3′ splice site, and 35 bp *Smndc1* intronic deletion. Red box indicates the *Smndc1* PE and arrows indicate guide RNA sequences. Blue dotted arrow in the *Smndc1* PE deletion allele indicates a 77 bp sequence originally downstream of the PE which was inverted in the genetically modified allele. **C**, Sanger sequence of *A*. *thaliana* lines with deletion of the *SPF30* PE, and *SPF30* intronic deletion. Green box indicates PE and arrows indicate guide RNA sequences.
(TIF)

S5 Fig *Smndc1* poison exon deletion increases transcript and protein abundance and impacts global splicing. **A**, Correlation between percent of *Smndc1* poison exon (PE) read coverage and total *Smndc1* transcript abundance across liver samples from wild-type (WT/WT), heterozygous (WT/Δ), and homozygous (Δ/Δ) mice. R, Pearson correlation coefficient. *n* = 17 animals. **B**, Western blot demonstrating SMNDC1 and alpha-tubulin signal and normalized ratio in liver lysate from wild-type and PE homozygous mice. *n* = 10 animals per genotype. **C**, Western blot demonstrating SMNDC1 and alpha-tubulin signal and normalized ratio in cerebellum lysate from wild-type and PE homozygous mice. *n* = 15 replicates per genotype. **D**, Quantification of SMNDC1 signal from Western blots generated with wild-type (WT/WT) and PE null (Δ/Δ) mouse cerebellum lysate. Signal normalized to alpha-tubulin. P-value computed using one-sided Student’s T-test. *n* = 15 replicates per genotype. **E-F**, Scatterplots of constitutive intron splicing (**C**) and tandem 3′ UTR reads (**D**) from RNA-sequencing of liver and fibroblast. Comparison between wild-type and *Smndc1* PE homozygous mice. Red and blue dots represent significantly increased and decreased spliced isoforms in the PE null samples, respectively. *n* = 5 biological replicates per genotype.
(TIF)

S6 Fig *Smndc1* poison exon deletion is associated with altered expression of genes involved in metabolism and RNA processing. **A**, Venn diagram of the overlapping MSigDB Hallmark pathways differentially spliced in murine fibroblast, cerebellum, and liver samples. **B**, GSEA pathway enrichment from liver RNA-seq. Pathways enriched in *Smndc1* poison exon (PE) null samples include ncRNA metabolic processing (Normalized Enrichment Score (NES) = 2.13; *p* = 0.0005), RNA processing (NES = 1.93; *p* = 0.0043), rRNA metabolic processing (NES = 2.07; *p* = 0.0011), and RNA metabolic processing (NES = 1.59; *p* = 0.030). Enrichment score for each gene set normalized to size of gene set to determine NES. Benjamini-Hochberg adjusted *p* values. *n* = 5 biological replicates per genotype. **C**, RNA-seq expression (TPM) from wild-type liver samples (WT/WT, *n* = 7 biological replicates) and *Smndc1* PE null liver samples (Δ/Δ, *n* = 6 biological replicates). Statistical significance was assessed by one-sided Wilcoxon-rank sum test.
(TIF)

S7 Fig *SMNDC1* knock down reverts key splicing phenotypes caused by *SMNDC1* poison exon loss. **A**, Western blot demonstrating SMNDC1 signal from HeLa inducible Cas9 (HeLa-iCas9) cells with stable integration of paired guide RNAs (pgRNA) targeting control (*AAVS1*) or the *SMNDC1* poison exon (PE). Cell lines treated with either control (NTC) siRNA or two unique siRNAs targeting coding regions within *SMNDC1* (siRNA1 and siRNA2). **B**, Total *SMNDC1* mRNA abundance (TPM) from the same cell lines and treatments as panel (**A**). **C-D**, Scatterplot of constitutive intron (**C**) and cassette exon (**D**) splicing from HeLa-iCas9 clones expressing control (NTC, *AAVS1*) or *SMNDC1* PE-targeting pgRNAs (pgSMNDC1). Red and blue points represent increased and decreased constitutive intron excision (**C**) or increased and decreased cassette exon inclusion (**D**). **E-F**, as in **C-D**, with comparison of HeLa-iCas9 pgSMNDC1 cells treated with control (NTC) siRNA or siRNA1 targeting *SMNDC1* (10 μM). **G-H**, as in **C-D**, with comparison of HeLa-iCas9 pgSMNDC1 cells treated with control (NTC) siRNA or siRNA2 targeting *SMNDC1* (10 μM).
(TIF)

S8 Fig Generation of *Smndc1* knockout mouse model suggests essentiality of SMNDC1. **A**, Paired guide RNAs (pgRNAs, arrows) were designed to disrupt the exon 4 (gray box) of *Smndc1*. **B**, Sanger sequencing trace of 5 bp deletion allele at exon 4 (gray box), including pgRNA-targeted sequences (arrows). **C**, Representative Western blot of SMNDC1 signal from wild-type (Smndc1^+/+^) and heterozygous (Smndc1^+/-^) mouse liver lysate, normalized to alpha-tubulin signal. *n* = 2 animals per genotype. **D**, Mendelian analysis of genotyped tissue from three week old offspring. Dotted lines represent normal genotypic percentages. P-values generated through Chi-squared testing.
(TIF)

S9 Fig *Smndc1* poison exon deletion does not impact litter composition, survival, or aging. **A**, Quantification of mean litter sizes born to intercrossed and backcrossed mice from the intronic deletion (blue) and combined *Smndc1* poison exon (PE) and 3′ splice site deletion (red) lines. Error bars represent +/- standard error. Statistic computed using two-sided Wilcoxon rank-sum; *n* = 54 litters (intronic deletion line) and 116 litters (combined poison exon and 3′ splice site deletion lines). **B**, Proportional representation of male and female pup births across deletion lines as in (**A**). *P*-value computed using chi-squared test. **C**, Kaplan-Meier survival curve of mice either wild-type (WT/WT), heterozygous (WT/Δ), or homozygous (Δ/Δ) for *Smndc1* PE (*n* = 30 total, both male and female mice). **D-E**, Weight quantification during aging for female (**D**, *n* = 27) and male mice (**E**, *n* = 9) of either WT/WT or PE Δ/Δ genotype. Boxplot upper whisker extends to the largest value no further than 1.5 * IQR.
(TIF)

S10 Fig Proliferative and necrotizing polyarteritis in *Smndc1* poison exon null mouse. **A-E**, Hematoxylin and eosin staining of multiple tissues from a 6-month-old, male *Smndc1* poison exon null mouse. Imaging depicts arteritis lesions containing neutrophilic inflammation and necrosis, with more chronic intimal and medial proliferation, fibrosis, and lymphocytic inflammation. Lower-magnification of prostatic artery, artery of skull (presumed temporal artery), and thoracic aorta (**A-C**, respectively); objective lens 20X, scale bar: 100 μm. Higher-magnification of lumbar arteriole and tongue arteriole (**D-E**, respectively); objective lens 40X, scale bar: 50 μm.
(TIF)

S11 Fig Weight alteration in *Smndc1* poison exon null male mice. **A**, Quantification of homozygous male pup weight at postnatal weeks 1–8. Weight measured for all pups born to homozygous breeder pairs from both the *Smndc1* intronic deletion and *Smndc1* poison exon deletion lines. Statistical significance was assessed by the Wilcoxon-rank sum test (*n* = 61 mice).
(TIF)

S1 Appendix Supporting mouse histology. (PDF)
