# Supplementary figures and images for: An autoregulatory poison exon in Smndc1 is conserved across kingdoms and influences organism growth
Source: PLoS Genet. 2024 Aug 16;20(8):e1011363. doi: 10.1371/journal.pgen.1011363 (PMC11357089; doi:10.1371/journal.pgen.1011363)

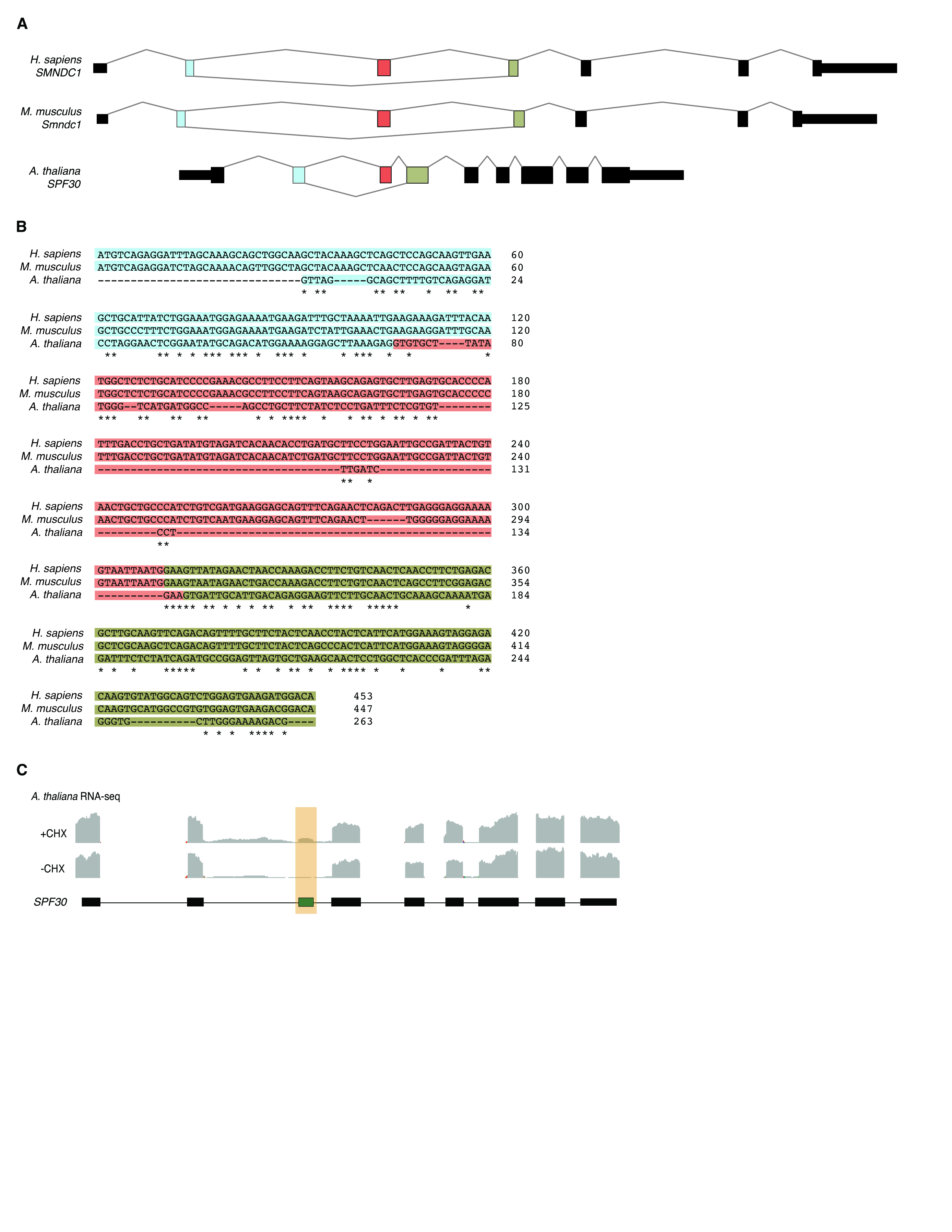

Supplement: S1 Fig — A, Schematic representation of the intron-exon structure of SMNDC1, Smndc1, and SPF30, respectively. Introns and exon lengths are scaled appropriately to each gene, but genes are not scaled to each other. Highlighted are upstream coding exons (blue), poison exons (red), and downstream coding exons (green). B, Alignment of human, mouse, and A. thaliana DNA sequences from coding and poison exons of SMNDC1, Smndc1 and SPF30, respectively. Highlighted are upstream coding exon (blue), poison exon (red), and downstream coding exon (green). C, BAM coverage plot of SPF30 gene structure and mapped read coverage in samples treated with or without cycloheximide (CHX). Poison exon supporting reads highlighted in yellow. The poison exon (green box) was previously unannotated. (TIF) [file pgen.1011363.s014.tif]

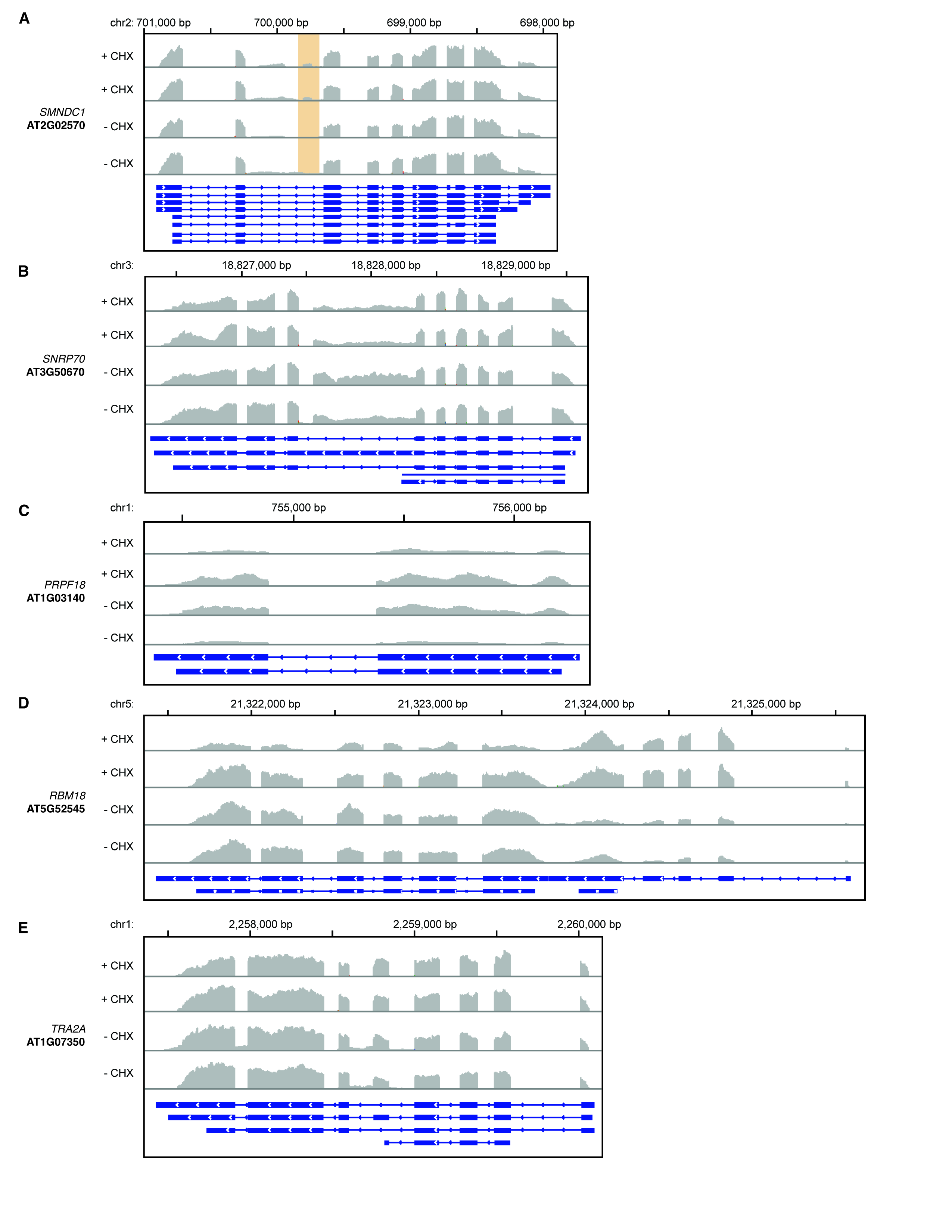

Supplement: S2 Fig — A-E, BAM coverage plots depicting transcript structure (blue) and mapped read coverage (gray) from samples treated with or without cycloheximide (+ CHX and–CHX, respectively). Poison exon supporting reads are highlighted in yellow. A, AT2G02570 (A. thaliana), an ortholog to SMNDC1 (human); B, AT3G50670 (A. thaliana), an ortholog to SNRP70 (human); C, AT1G03140 (A. thaliana), an ortholog to PRPF18 (human); D, AT5G52545 (A. thaliana), an ortholog to RBM18 (human); E, AT1G07350 (A. thaliana), an ortholog to TRA2A (human). (TIF) [file pgen.1011363.s015.tif]

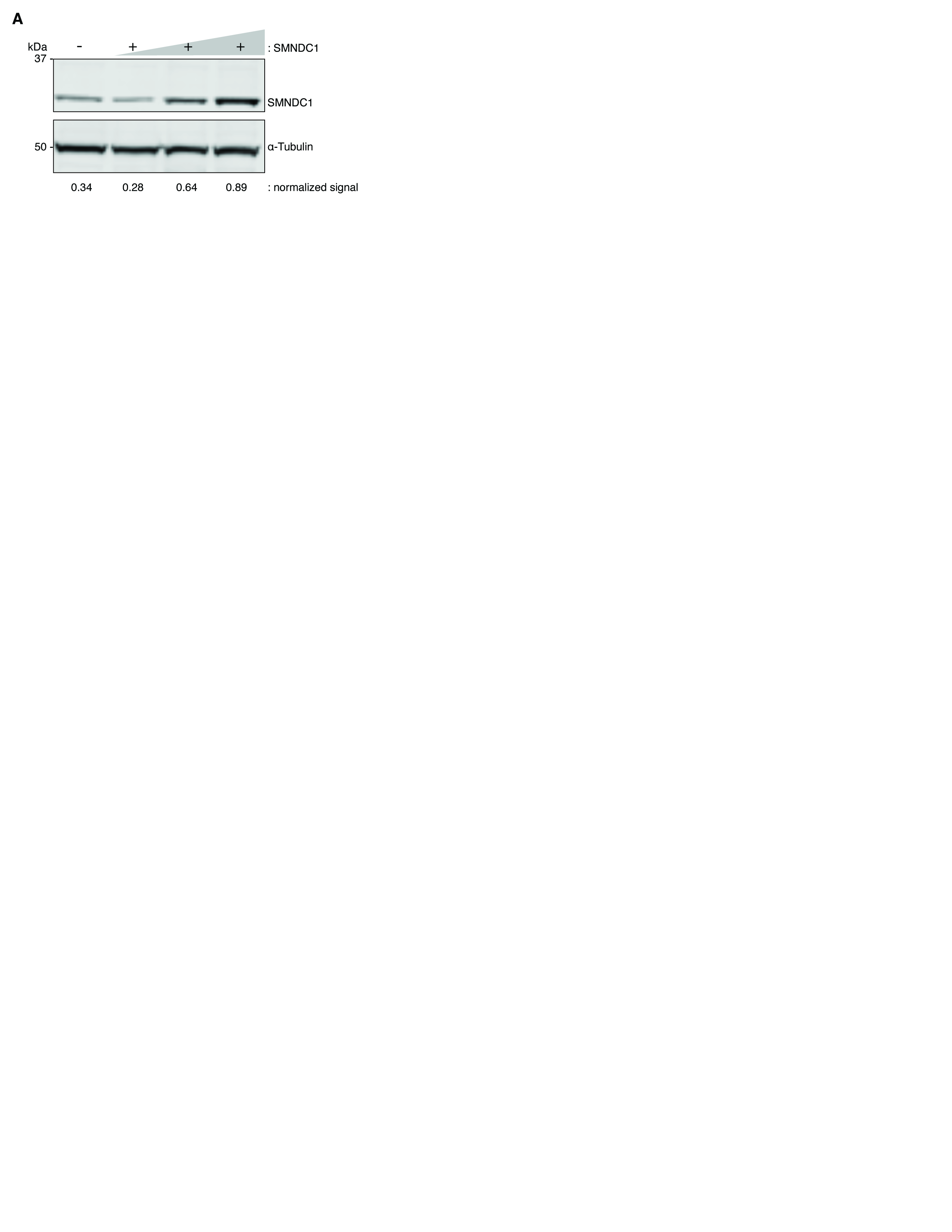

Supplement: S3 Fig — A, Western blot demonstrating SMNDC1 signal from mouse melanocytes using a transient overexpression system with increasing amounts of SMNDC1 expression. SMNDC1 signal is normalized to alpha-tubulin signal. (TIF) [file pgen.1011363.s016.tif]

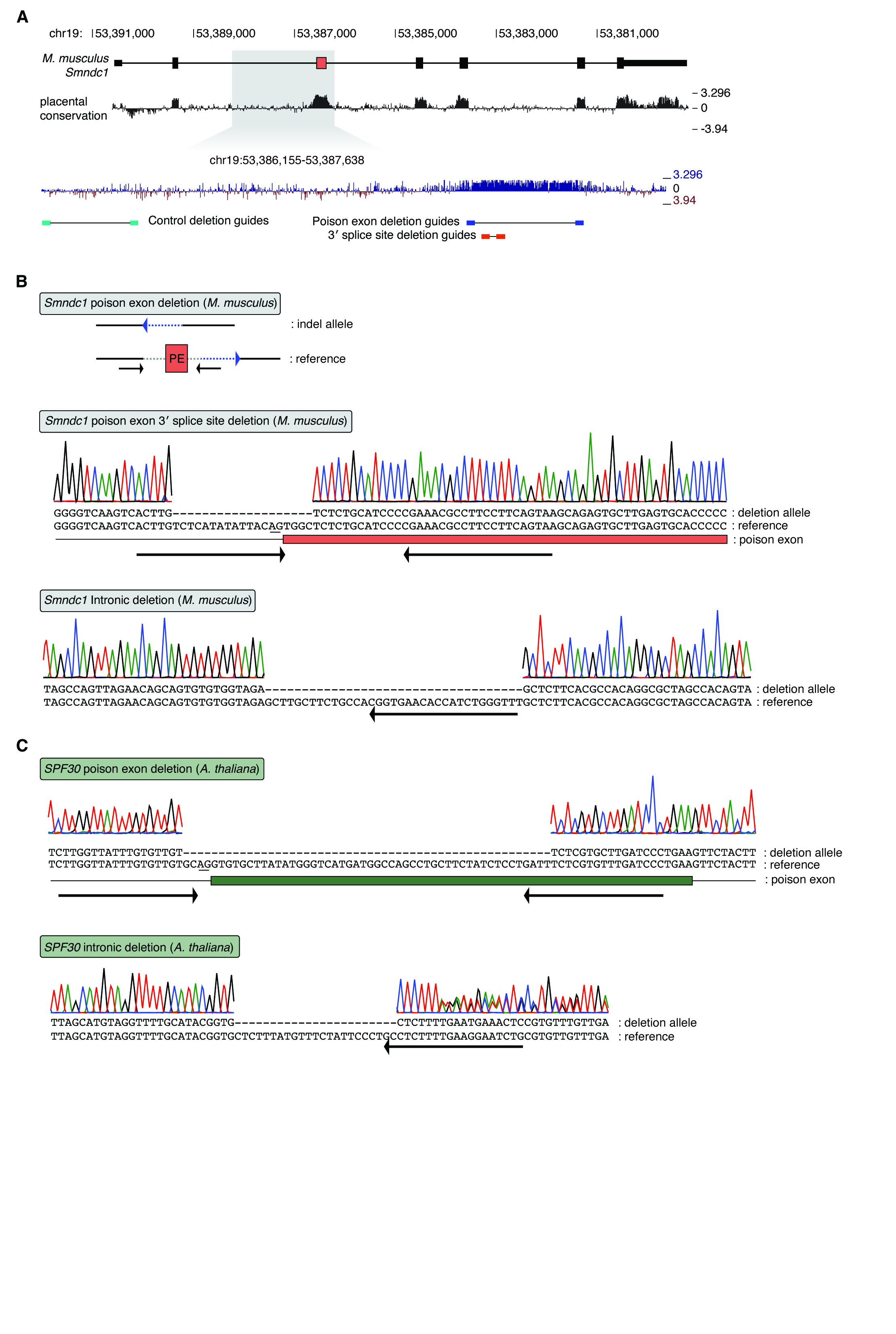

Supplement: S4 Fig — A, Paired guide RNAs (pgRNAs, colored boxes) designed to disrupt the Smndc1 poison exon (red box). pgRNAs target regions of high placental conservation or the upstream intronic region. B, Sanger sequencing of mouse lines with 254 base pair (bp) deletion of the Smndc1 poison exon (PE), 19 bp deletion of the Smndc1 PE 3′ splice site, and 35 bp Smndc1 intronic deletion. Red box indicates the Smndc1 PE and arrows indicate guide RNA sequences. Blue dotted arrow in the Smndc1 PE deletion allele indicates a 77 bp sequence originally downstream of the PE which was inverted in the genetically modified allele. C, Sanger sequence of A. thaliana lines with deletion of the SPF30 PE, and SPF30 intronic deletion. Green box indicates PE and arrows indicate guide RNA sequences. (TIF) [file pgen.1011363.s017.tif]

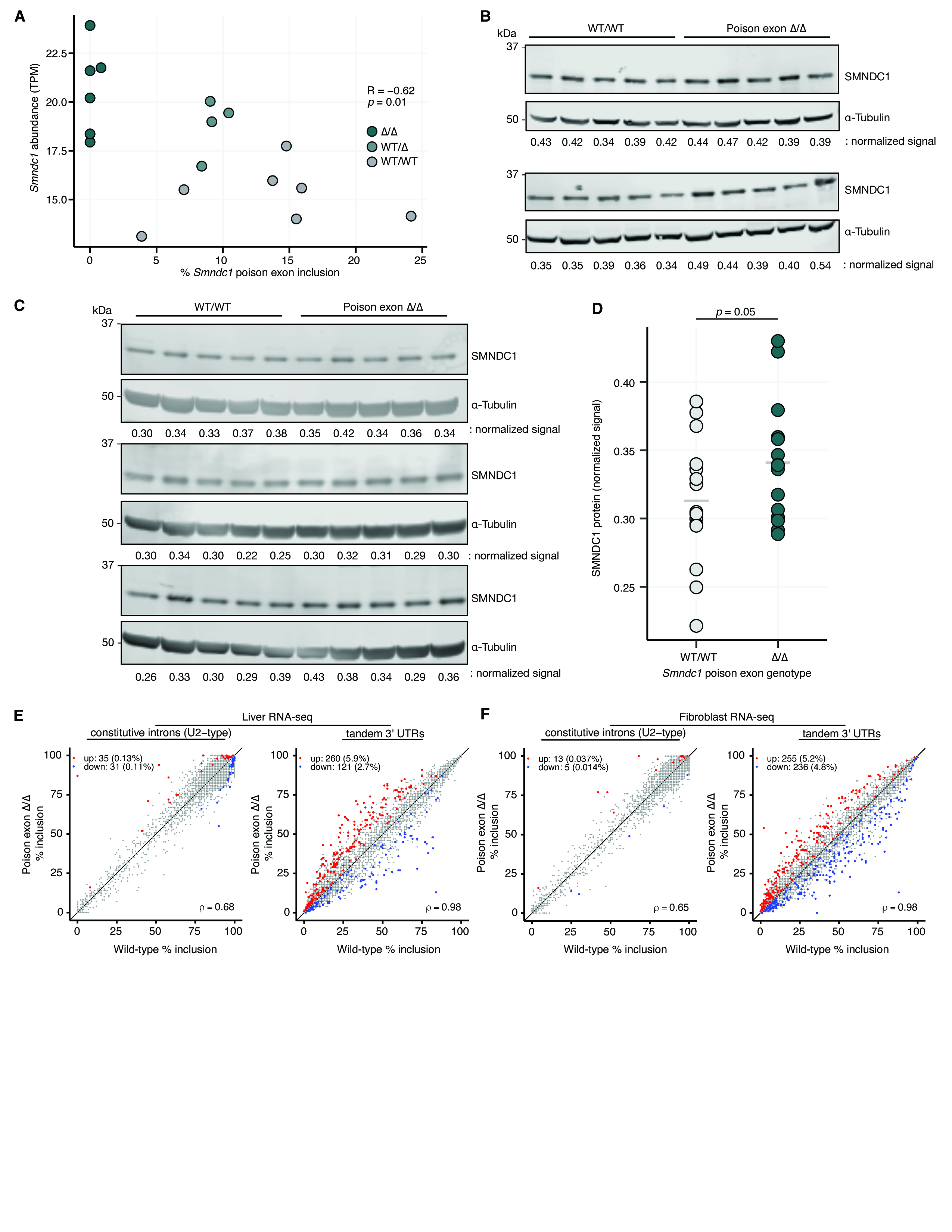

Supplement: S5 Fig — A, Correlation between percent of Smndc1 poison exon (PE) read coverage and total Smndc1 transcript abundance across liver samples from wild-type (WT/WT), heterozygous (WT/Δ), and homozygous (Δ/Δ) mice. R, Pearson correlation coefficient. n = 17 animals. B, Western blot demonstrating SMNDC1 and alpha-tubulin signal and normalized ratio in liver lysate from wild-type and PE homozygous mice. n = 10 animals per genotype. C, Western blot demonstrating SMNDC1 and alpha-tubulin signal and normalized ratio in cerebellum lysate from wild-type and PE homozygous mice. n = 15 replicates per genotype. D, Quantification of SMNDC1 signal from Western blots generated with wild-type (WT/WT) and PE null (Δ/Δ) mouse cerebellum lysate. Signal normalized to alpha-tubulin. P-value computed using one-sided Student’s T-test. n = 15 replicates per genotype. E-F, Scatterplots of constitutive intron splicing (C) and tandem 3′ UTR reads (D) from RNA-sequencing of liver and fibroblast. Comparison between wild-type and Smndc1 PE homozygous mice. Red and blue dots represent significantly increased and decreased spliced isoforms in the PE null samples, respectively. n = 5 biological replicates per genotype. (TIF) [file pgen.1011363.s018.tif]

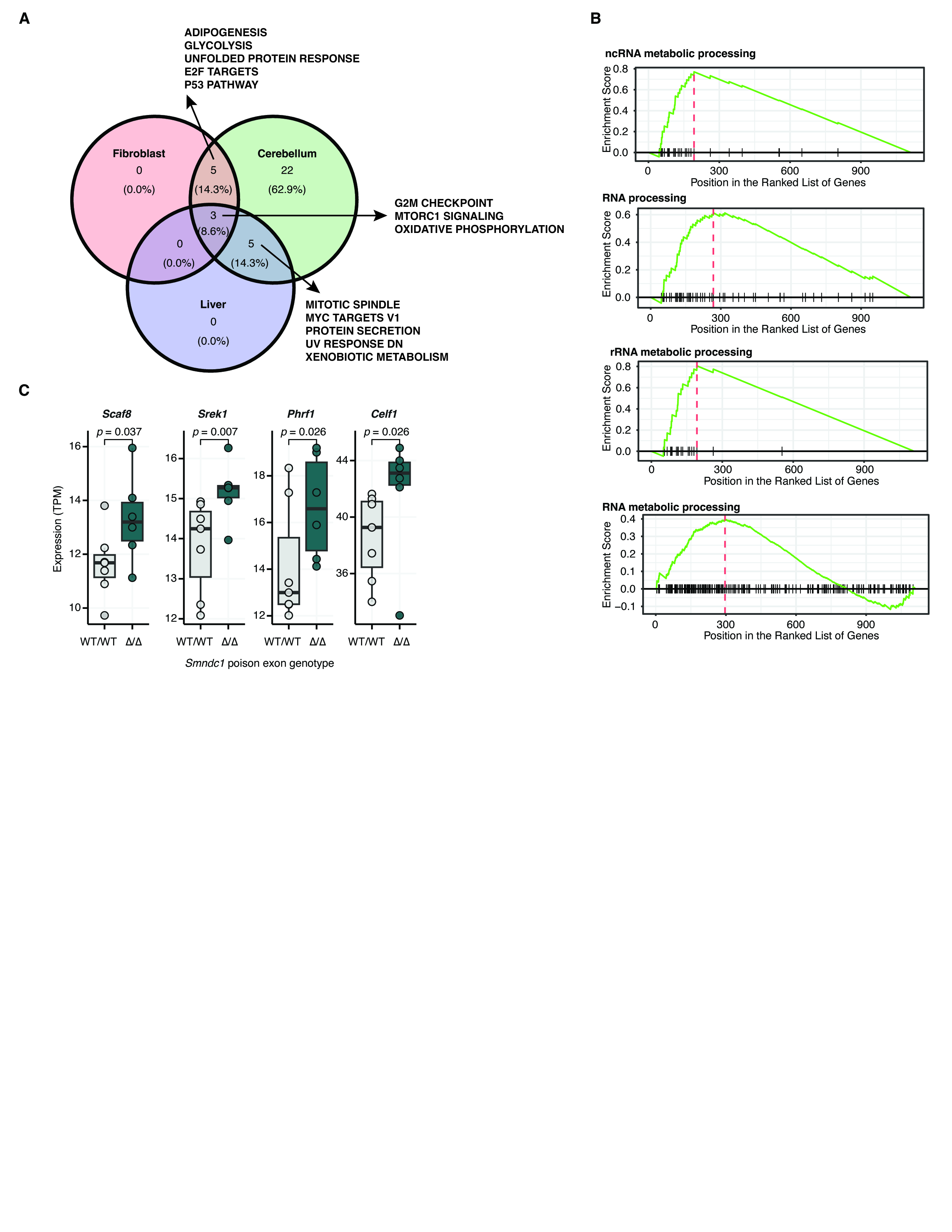

Supplement: S6 Fig — A, Venn diagram of the overlapping MSigDB Hallmark pathways differentially spliced in murine fibroblast, cerebellum, and liver samples. B, GSEA pathway enrichment from liver RNA-seq. Pathways enriched in Smndc1 poison exon (PE) null samples include ncRNA metabolic processing (Normalized Enrichment Score (NES) = 2.13; p = 0.0005), RNA processing (NES = 1.93; p = 0.0043), rRNA metabolic processing (NES = 2.07; p = 0.0011), and RNA metabolic processing (NES = 1.59; p = 0.030). Enrichment score for each gene set normalized to size of gene set to determine NES. Benjamini-Hochberg adjusted p values. n = 5 biological replicates per genotype. C, RNA-seq expression (TPM) from wild-type liver samples (WT/WT, n = 7 biological replicates) and Smndc1 PE null liver samples (Δ/Δ, n = 6 biological replicates). Statistical significance was assessed by one-sided Wilcoxon-rank sum test. (TIF) [file pgen.1011363.s019.tif]

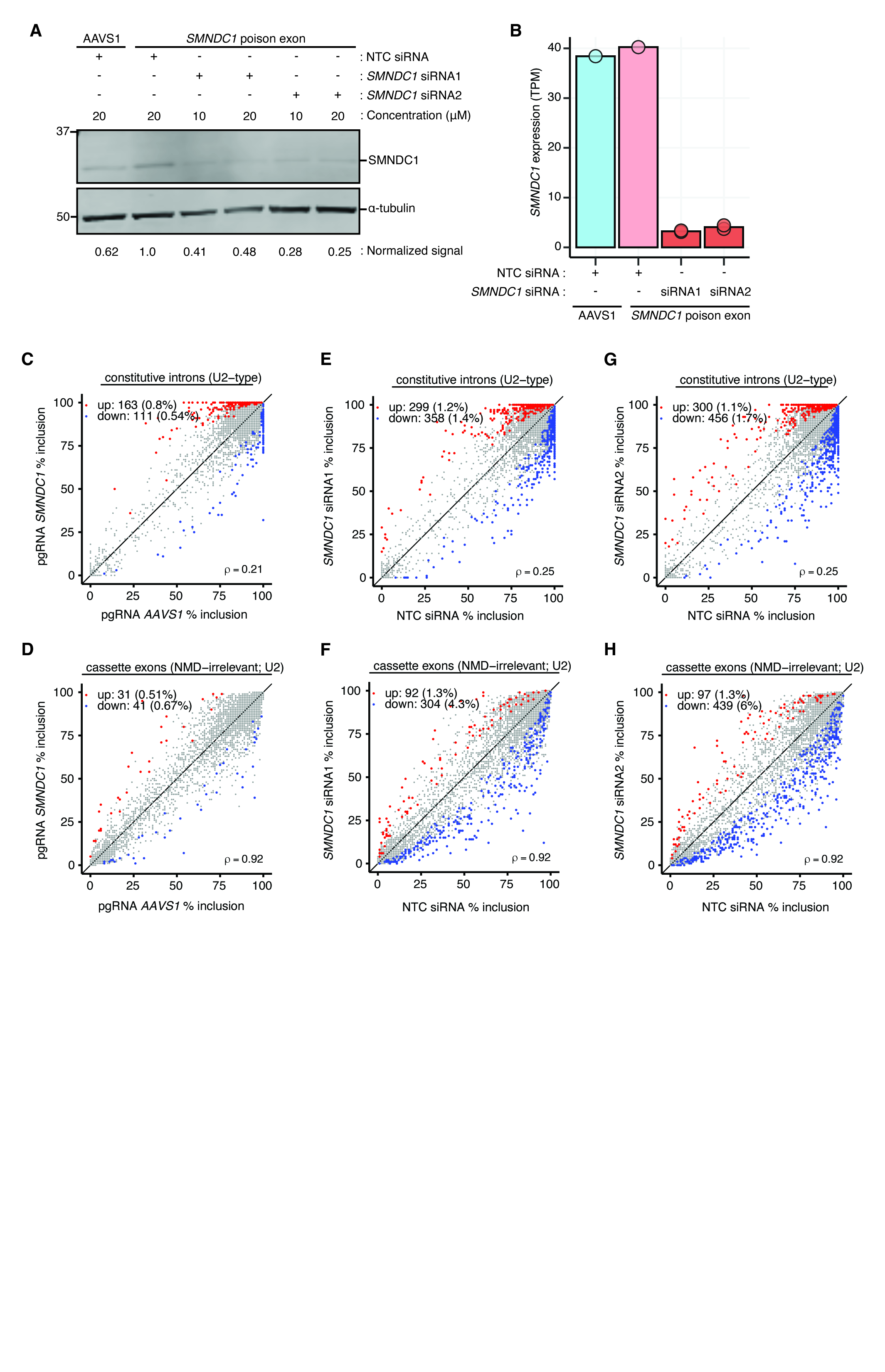

Supplement: S7 Fig — A, Western blot demonstrating SMNDC1 signal from HeLa inducible Cas9 (HeLa-iCas9) cells with stable integration of paired guide RNAs (pgRNA) targeting control (AAVS1) or the SMNDC1 poison exon (PE). Cell lines treated with either control (NTC) siRNA or two unique siRNAs targeting coding regions within SMNDC1 (siRNA1 and siRNA2). B, Total SMNDC1 mRNA abundance (TPM) from the same cell lines and treatments as panel (A). C-D, Scatterplot of constitutive intron (C) and cassette exon (D) splicing from HeLa-iCas9 clones expressing control (NTC, AAVS1) or SMNDC1 PE-targeting pgRNAs (pgSMNDC1). Red and blue points represent increased and decreased constitutive intron excision (C) or increased and decreased cassette exon inclusion (D). E-F, as in C-D, with comparison of HeLa-iCas9 pgSMNDC1 cells treated with control (NTC) siRNA or siRNA1 targeting SMNDC1 (10 μM). G-H, as in C-D, with comparison of HeLa-iCas9 pgSMNDC1 cells treated with control (NTC) siRNA or siRNA2 targeting SMNDC1 (10 μM). (TIF) [file pgen.1011363.s020.tif]

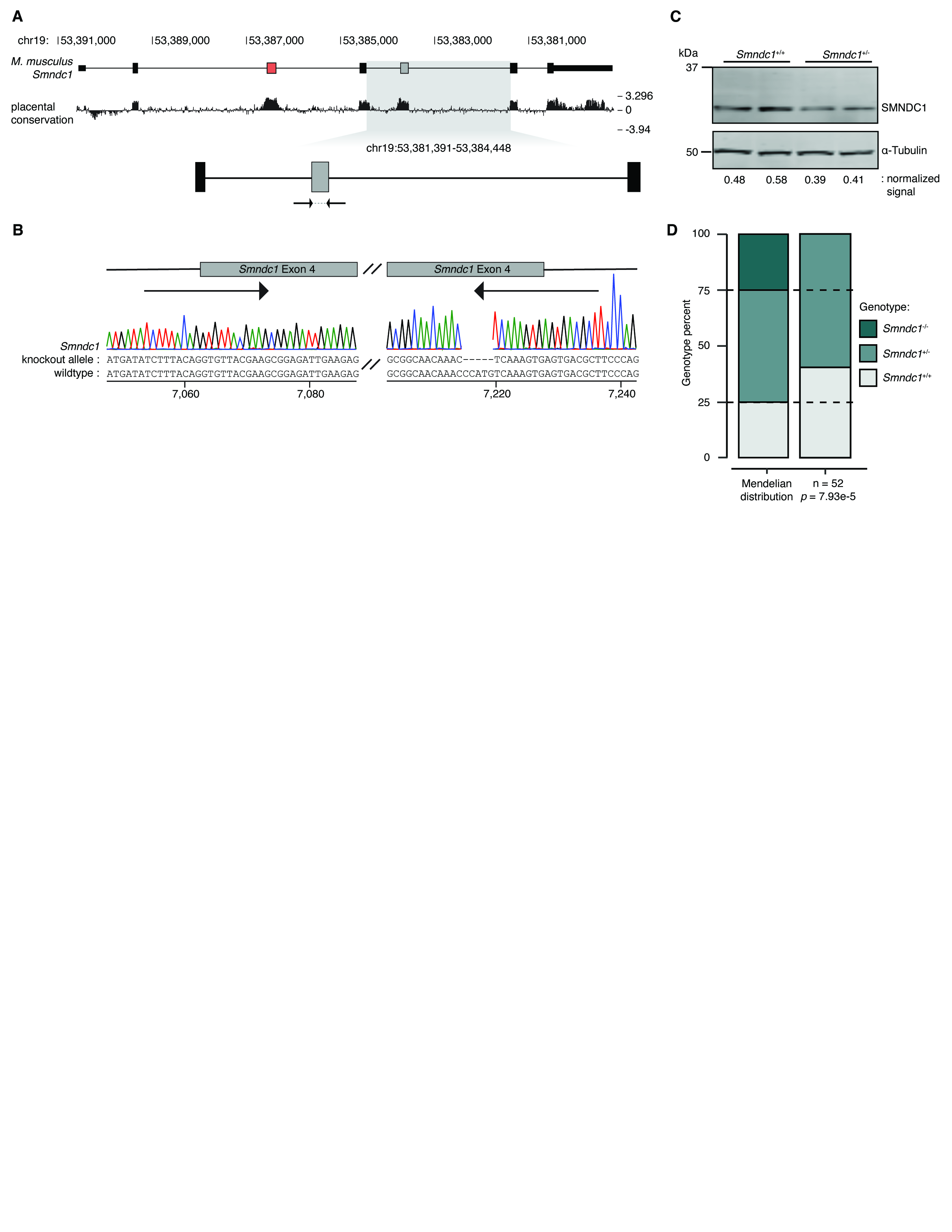

Supplement: S8 Fig — A, Paired guide RNAs (pgRNAs, arrows) were designed to disrupt the exon 4 (gray box) of Smndc1. B, Sanger sequencing trace of 5 bp deletion allele at exon 4 (gray box), including pgRNA-targeted sequences (arrows). C, Representative Western blot of SMNDC1 signal from wild-type (Smndc1+/+) and heterozygous (Smndc1+/-) mouse liver lysate, normalized to alpha-tubulin signal. n = 2 animals per genotype. D, Mendelian analysis of genotyped tissue from three week old offspring. Dotted lines represent normal genotypic percentages. P-values generated through Chi-squared testing. (TIF) [file pgen.1011363.s021.tif]

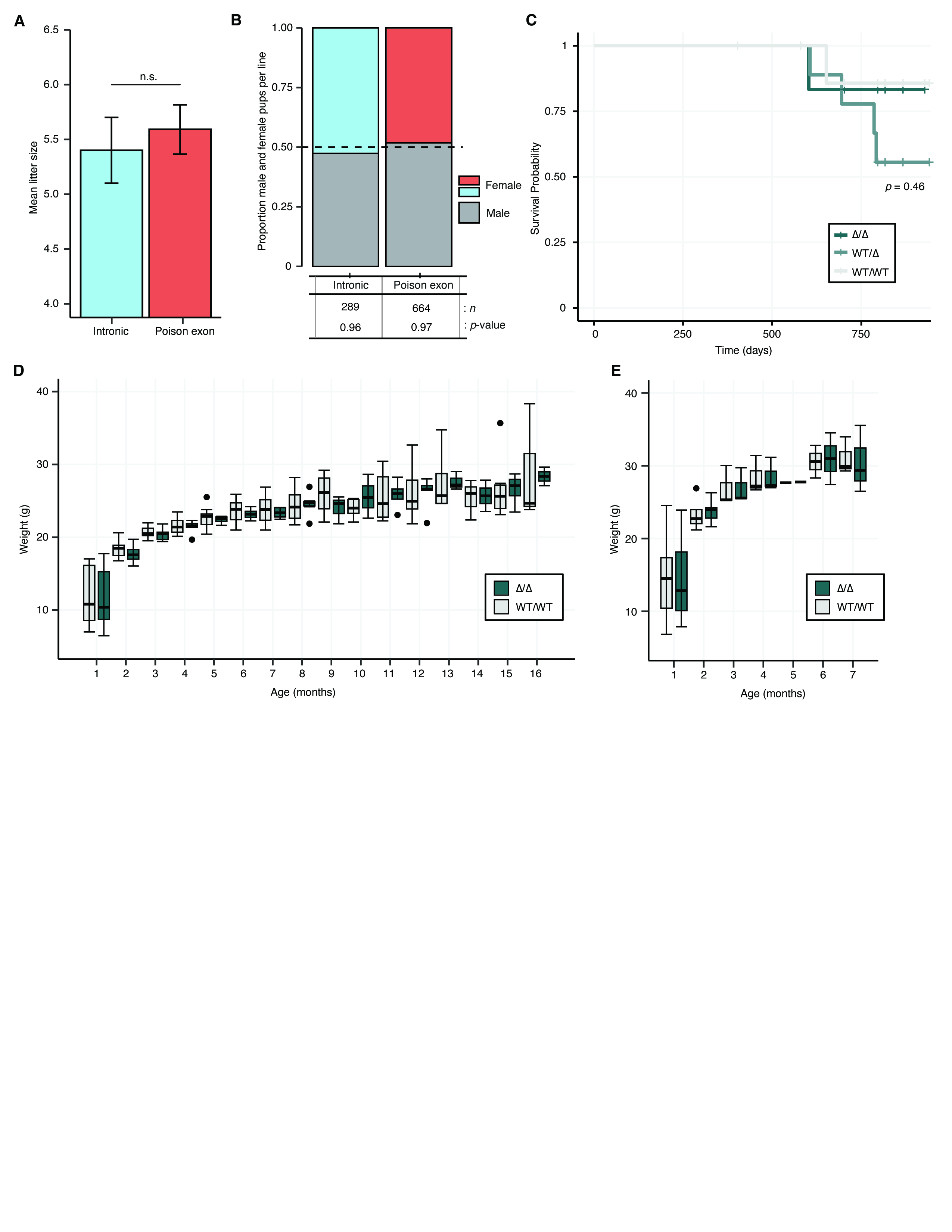

Supplement: S9 Fig — A, Quantification of mean litter sizes born to intercrossed and backcrossed mice from the intronic deletion (blue) and combined Smndc1 poison exon (PE) and 3′ splice site deletion (red) lines. Error bars represent +/- standard error. Statistic computed using two-sided Wilcoxon rank-sum; n = 54 litters (intronic deletion line) and 116 litters (combined poison exon and 3′ splice site deletion lines). B, Proportional representation of male and female pup births across deletion lines as in (A). P-value computed using chi-squared test. C, Kaplan-Meier survival curve of mice either wild-type (WT/WT), heterozygous (WT/Δ), or homozygous (Δ/Δ) for Smndc1 PE (n = 30 total, both male and female mice). D-E, Weight quantification during aging for female (D, n = 27) and male mice (E, n = 9) of either WT/WT or PE Δ/Δ genotype. Boxplot upper whisker extends to the largest value no further than 1.5 * IQR. (TIF) [file pgen.1011363.s022.tif]

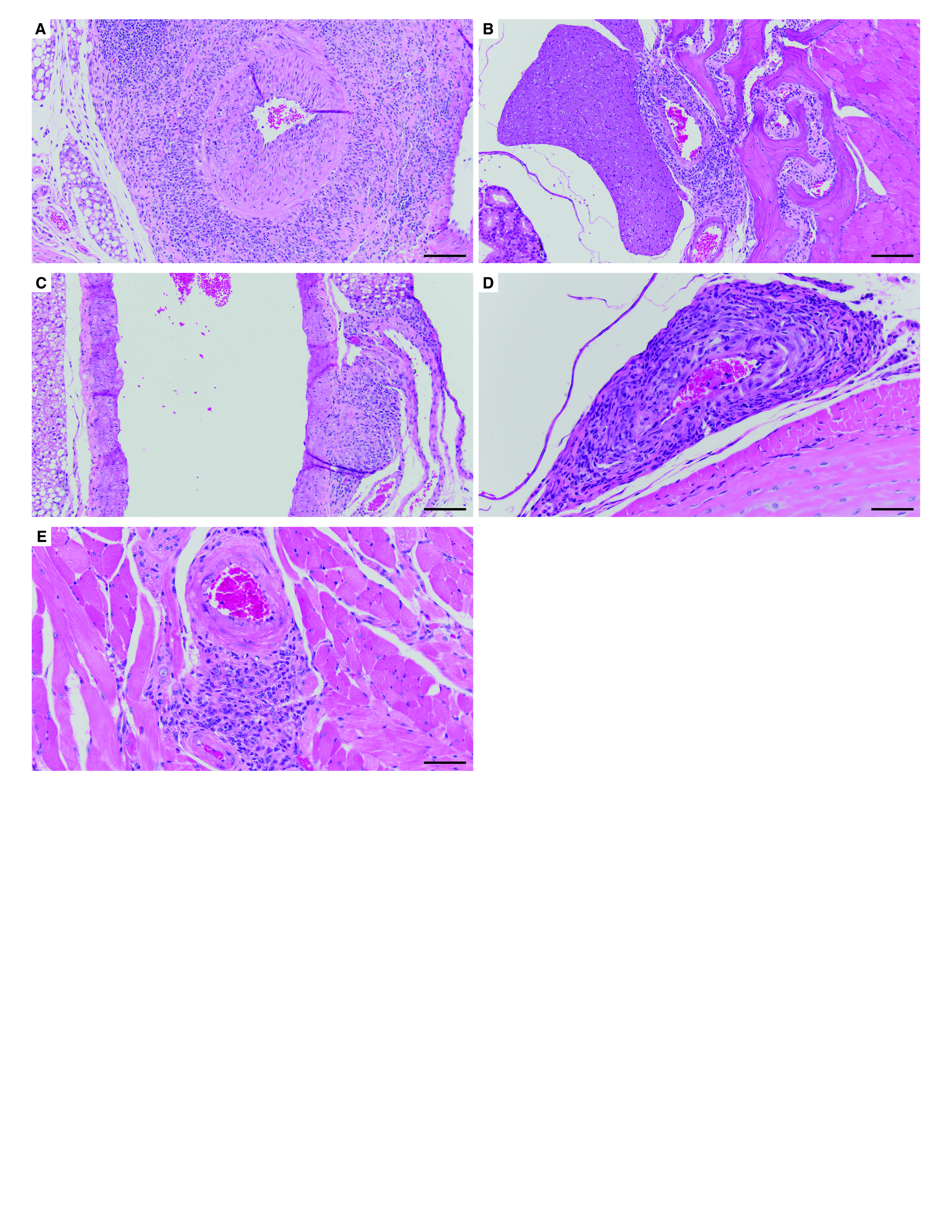

Supplement: S10 Fig — A-E, Hematoxylin and eosin staining of multiple tissues from a 6-month-old, male Smndc1 poison exon null mouse. Imaging depicts arteritis lesions containing neutrophilic inflammation and necrosis, with more chronic intimal and medial proliferation, fibrosis, and lymphocytic inflammation. Lower-magnification of prostatic artery, artery of skull (presumed temporal artery), and thoracic aorta (A-C, respectively); objective lens 20X, scale bar: 100 μm. Higher-magnification of lumbar arteriole and tongue arteriole (D-E, respectively); objective lens 40X, scale bar: 50 μm. (TIF) [file pgen.1011363.s023.tif]

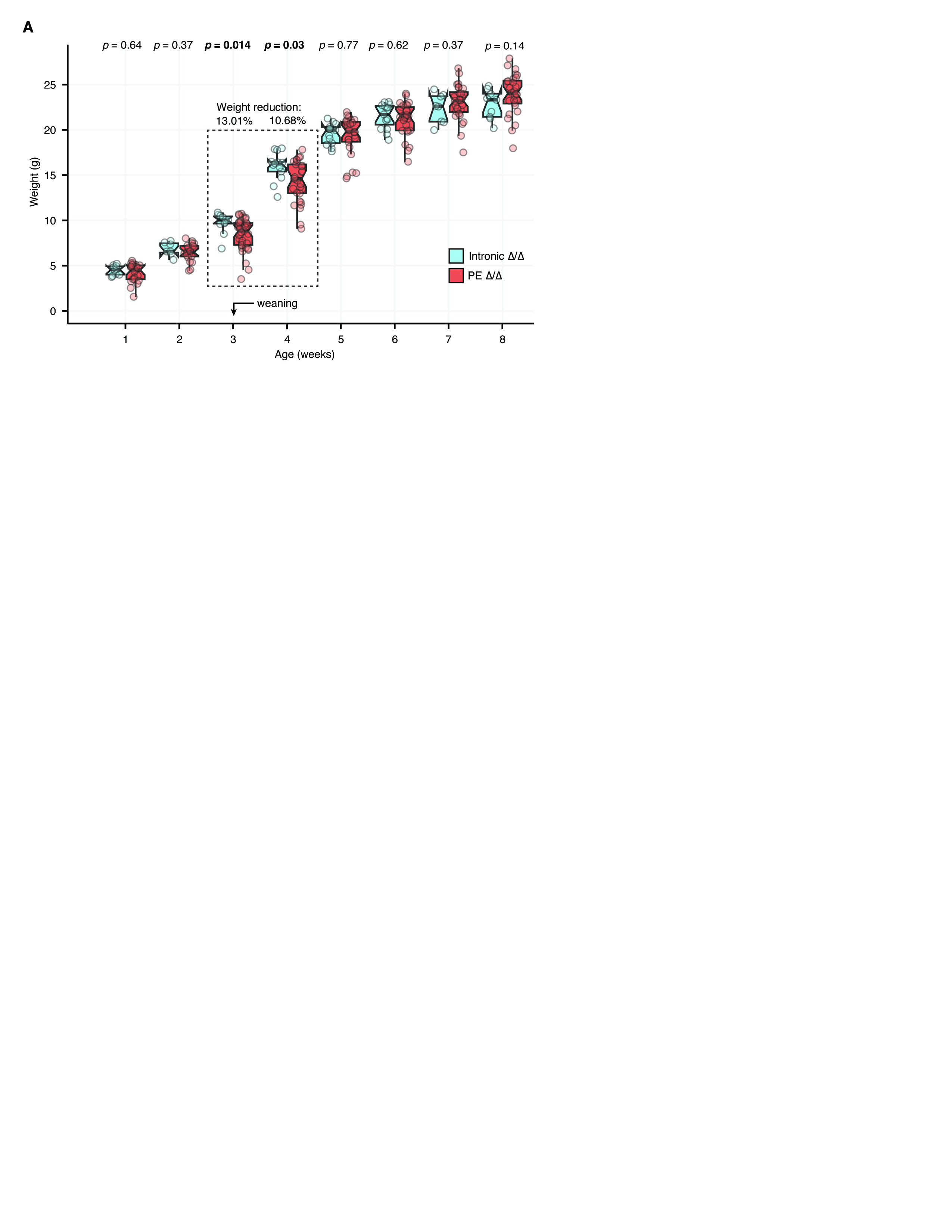

Supplement: S11 Fig — A, Quantification of homozygous male pup weight at postnatal weeks 1–8. Weight measured for all pups born to homozygous breeder pairs from both the Smndc1 intronic deletion and Smndc1 poison exon deletion lines. Statistical significance was assessed by the Wilcoxon-rank sum test (n = 61 mice). (TIF) [file pgen.1011363.s024.tif]
