# Supplementary material for: An autoregulatory poison exon in Smndc1 is conserved across kingdoms and influences organism growth
Source: PLoS Genet. 2024 Aug 16;20(8):e1011363. doi: 10.1371/journal.pgen.1011363 (PMC11357089; doi:10.1371/journal.pgen.1011363)

Supplementary Appendix 1: Supporting mouse histology.

220309_2.brain

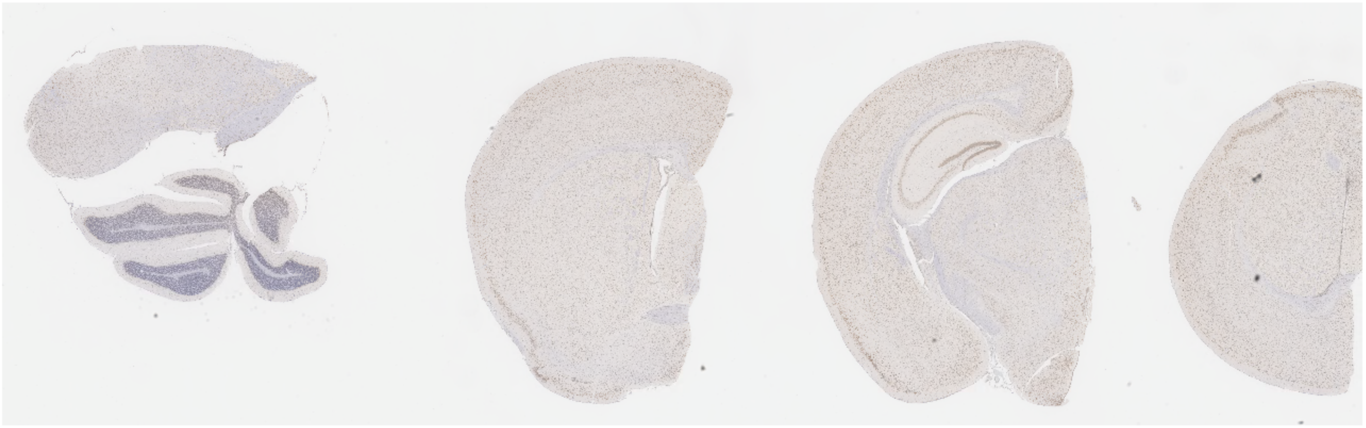

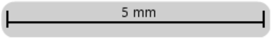

220309_2.lung

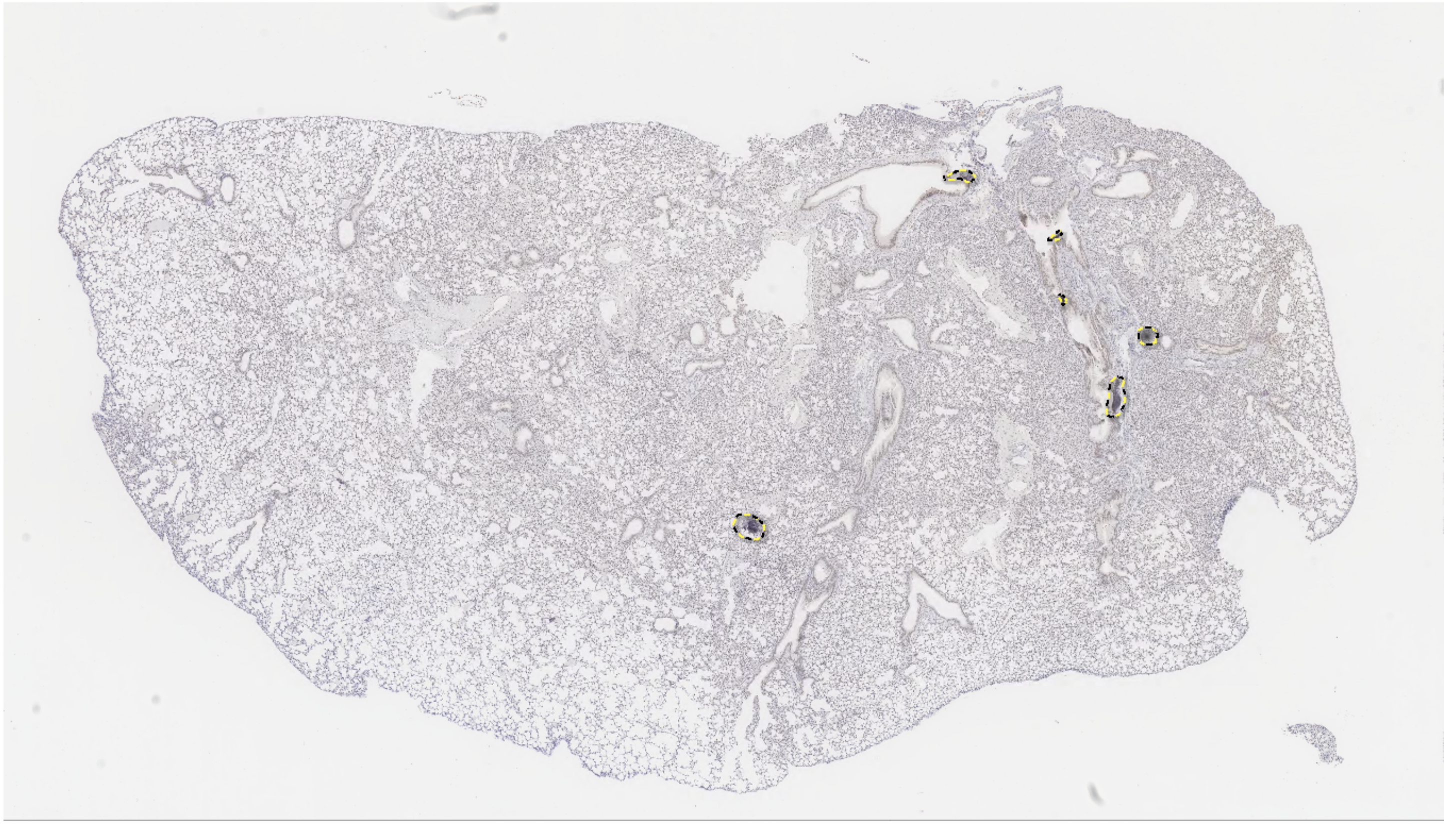

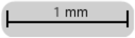

220309_2.quadriceps

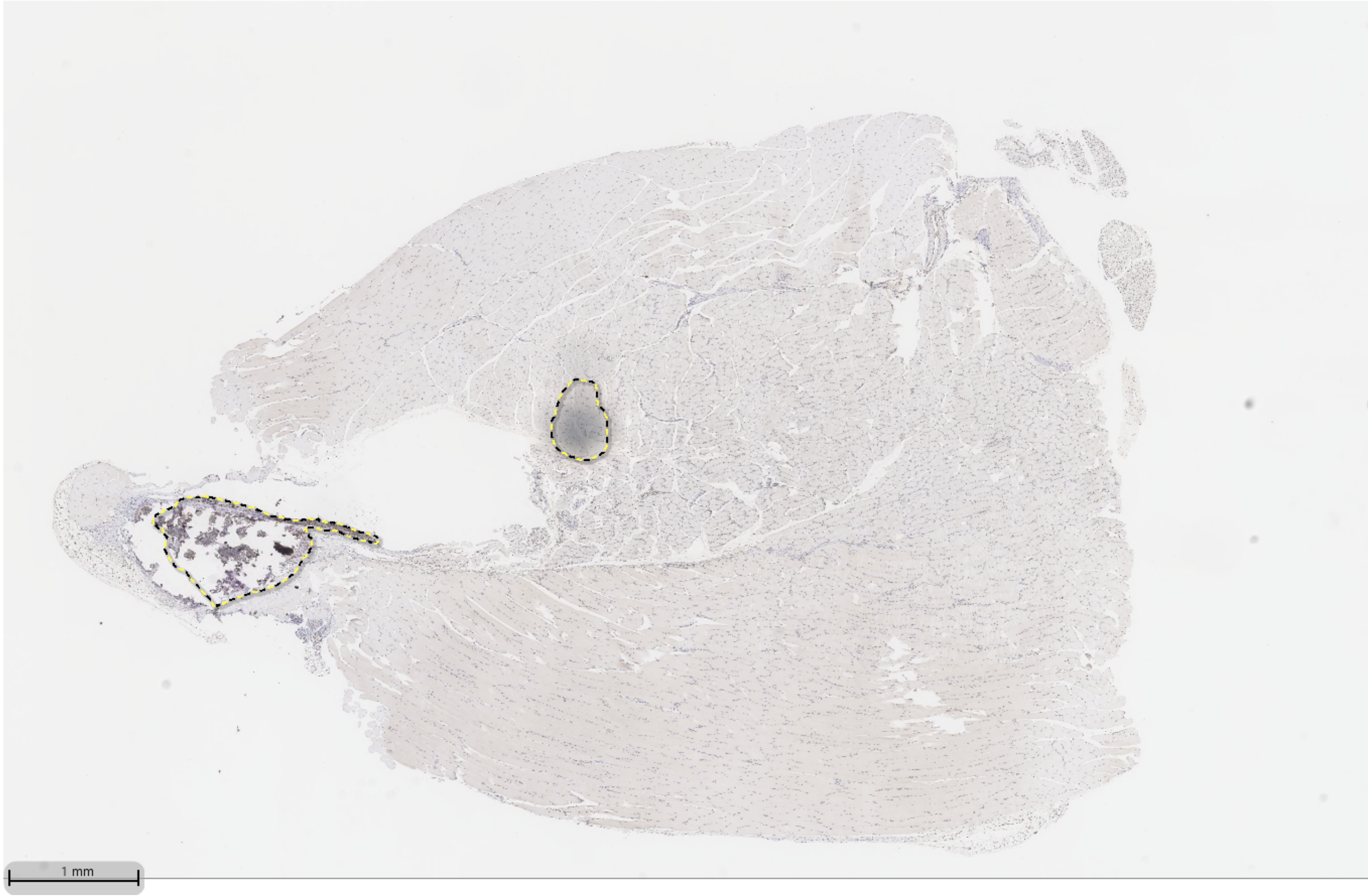

220309_3.brain

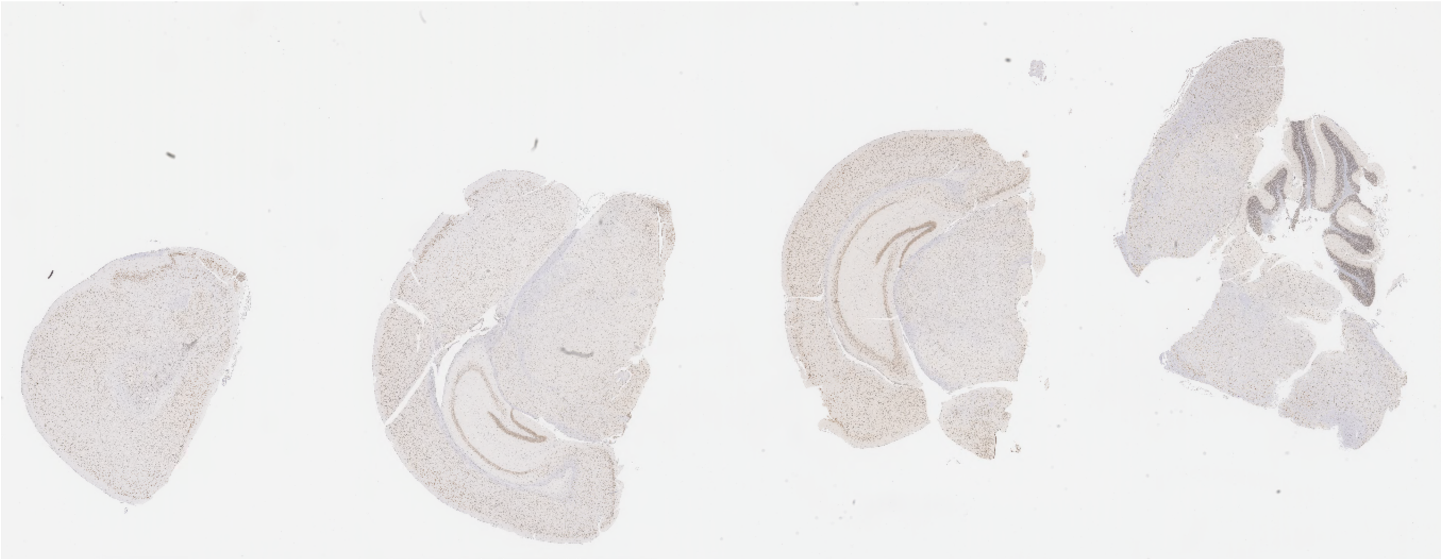

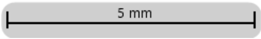

220309_3.lung

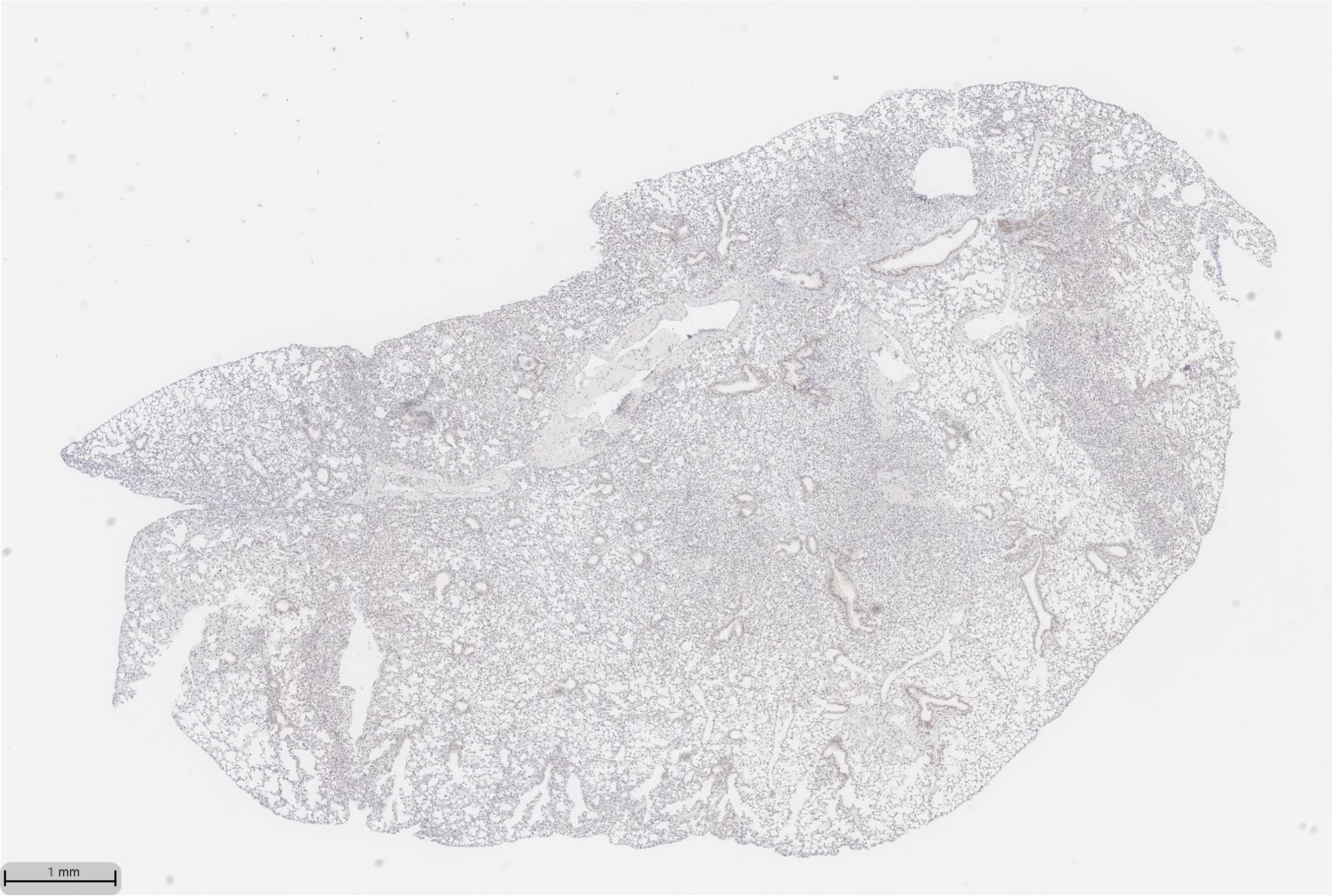

220309_3.quadriceps

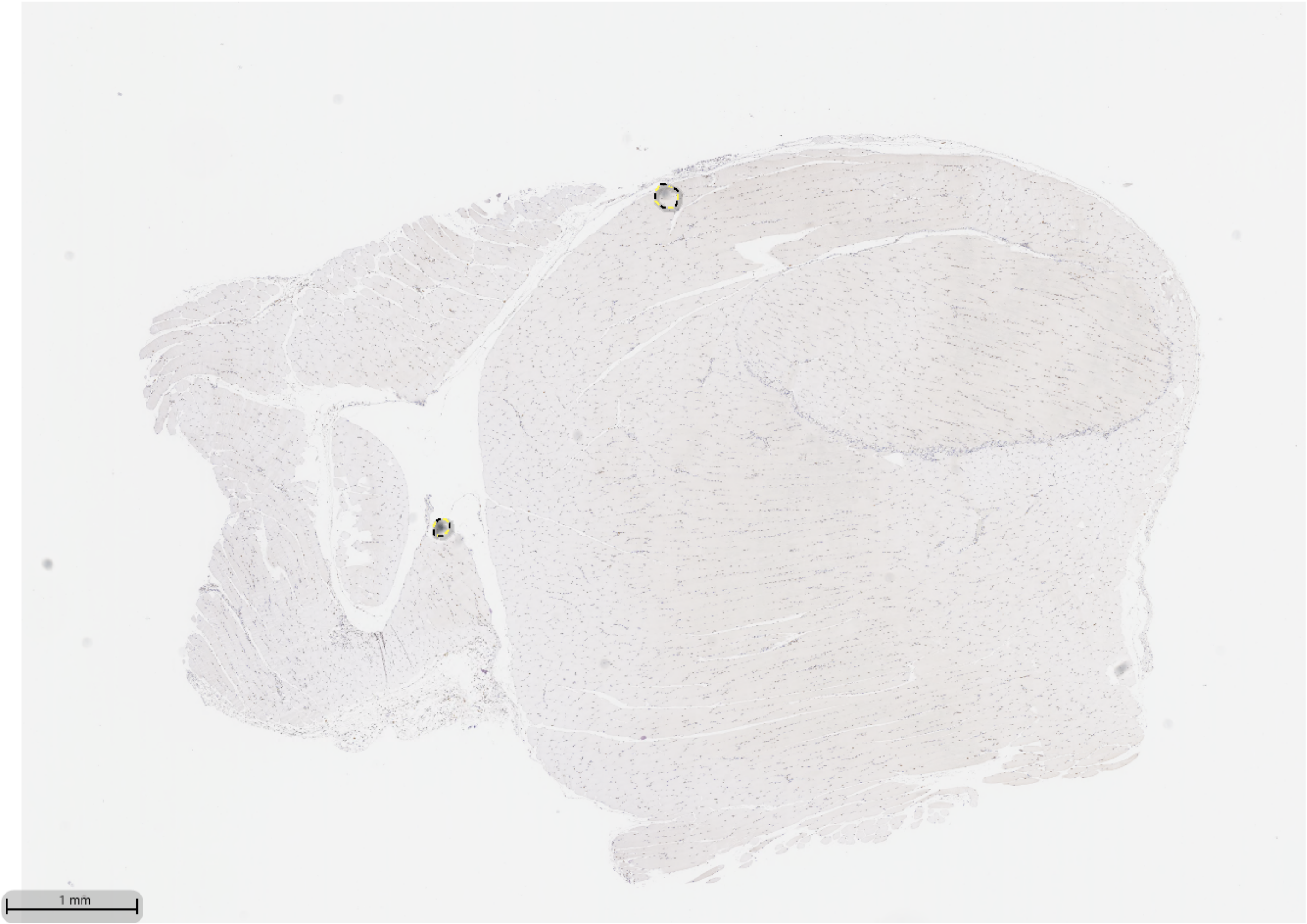

220524_2.brain

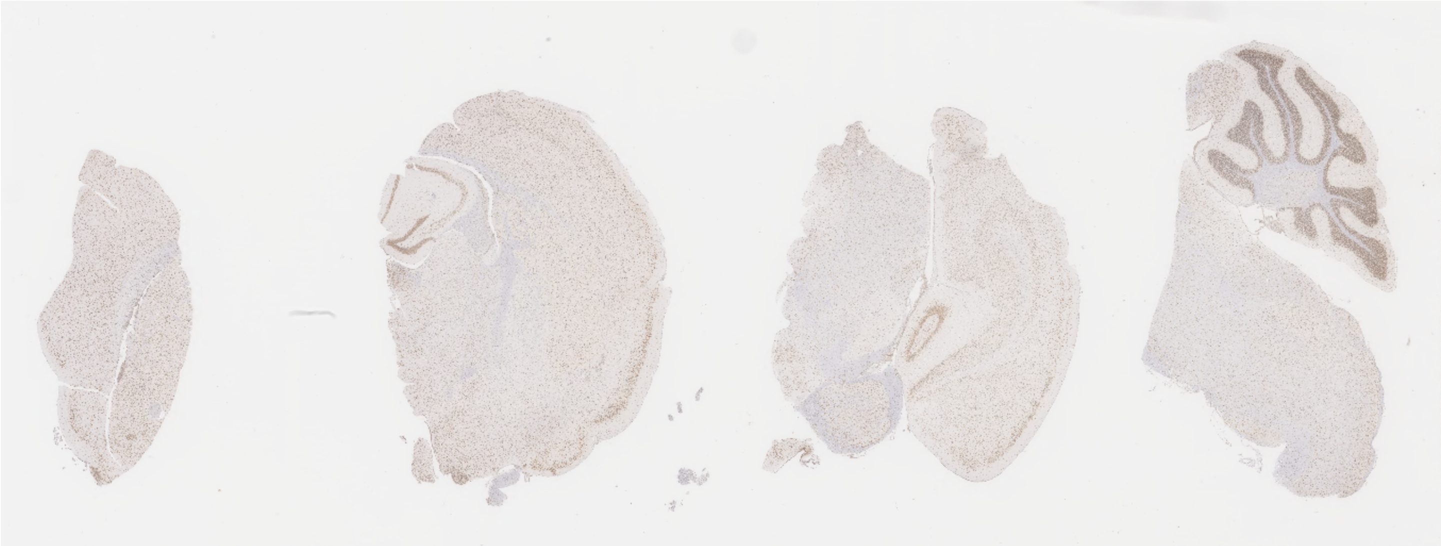

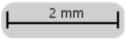

220524_2.lung

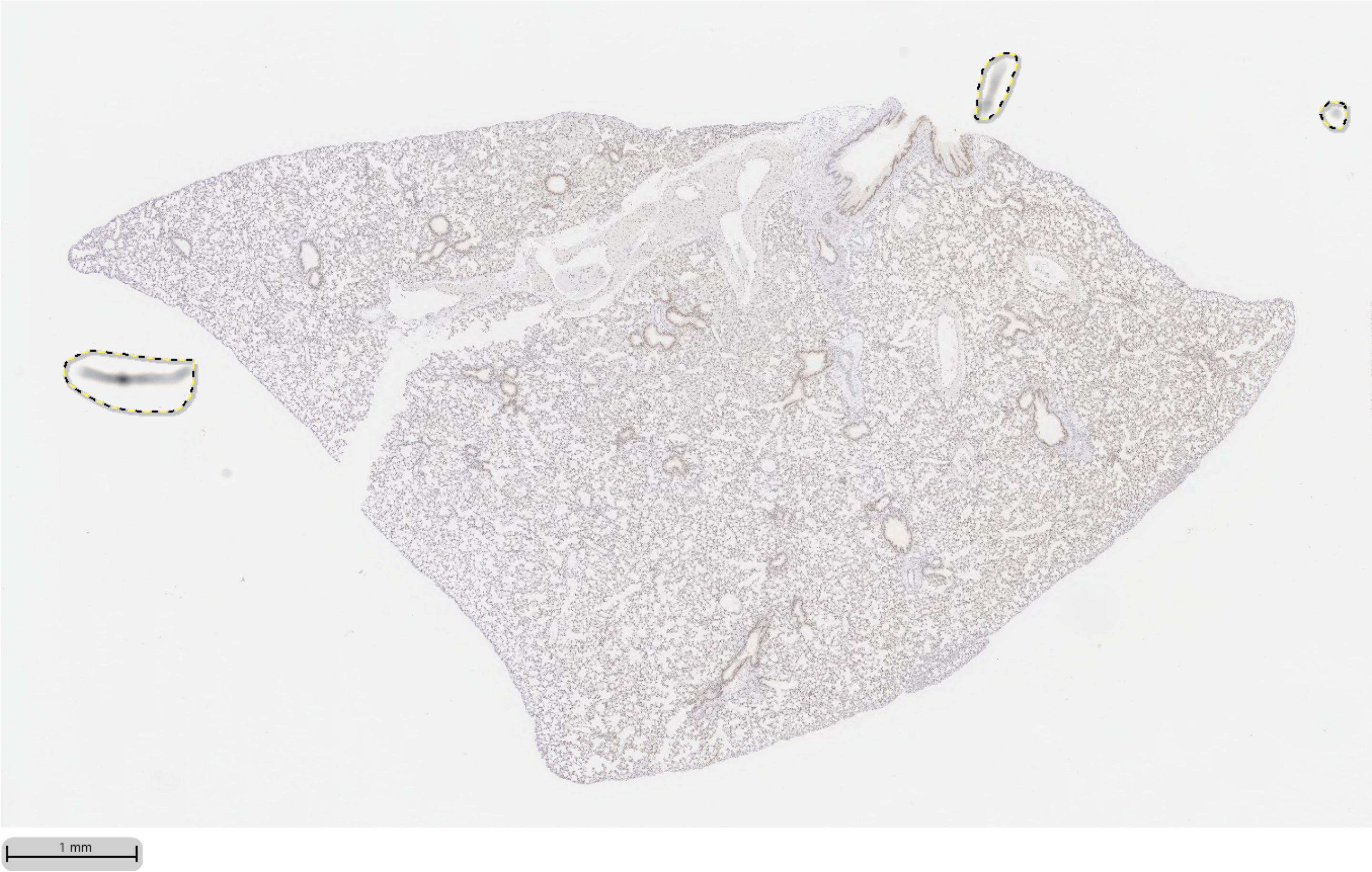

220524_2.quadriceps

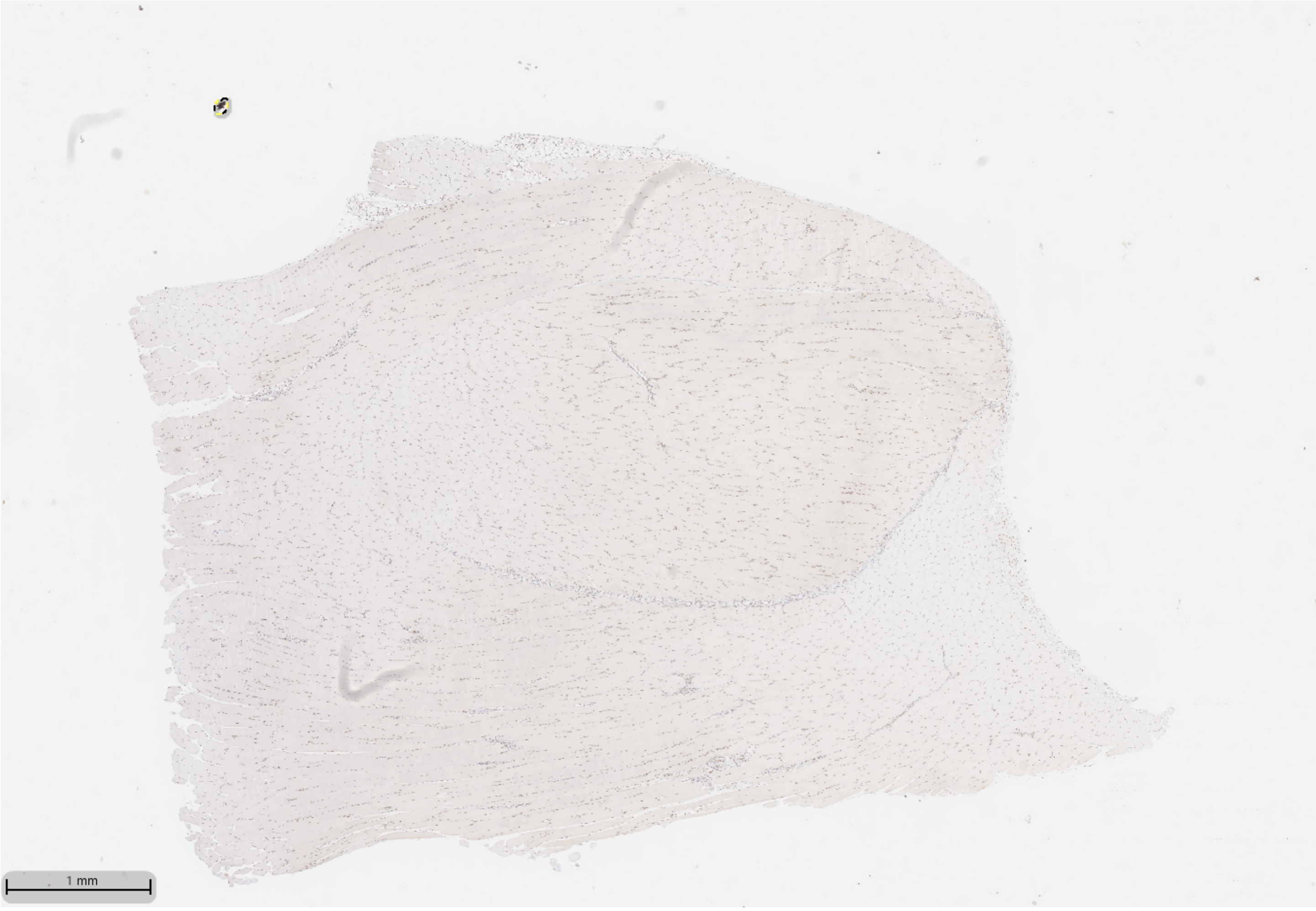

220524_3.brain

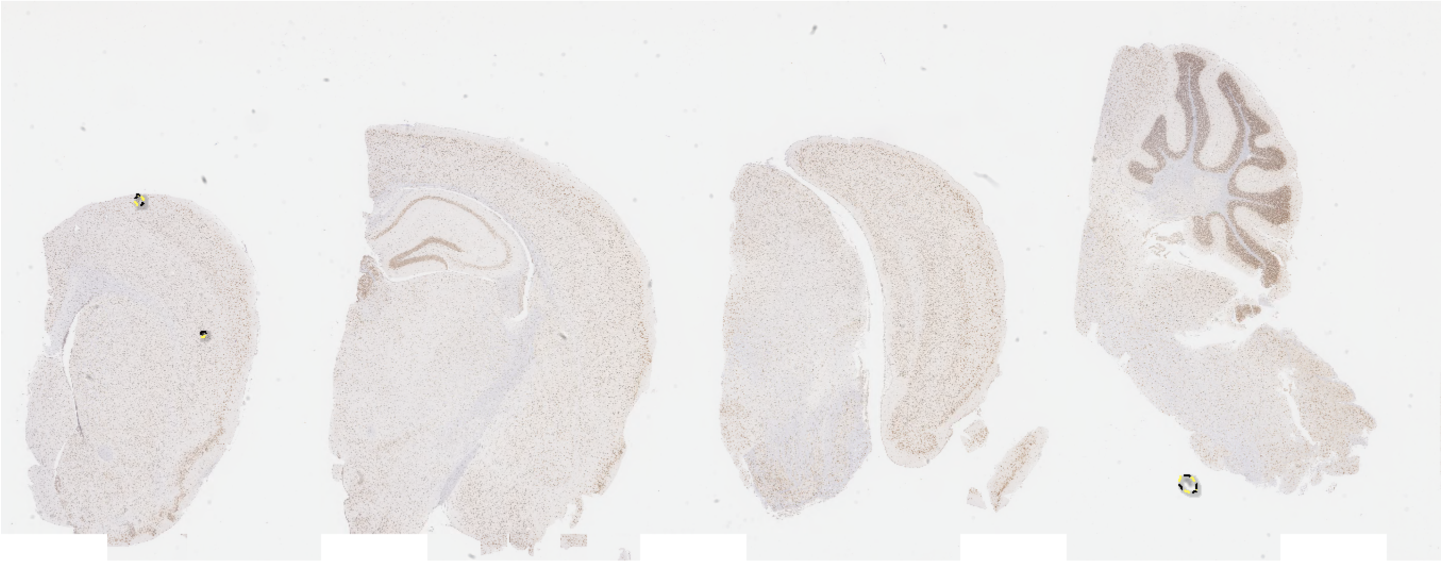

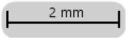

220524_3.lung

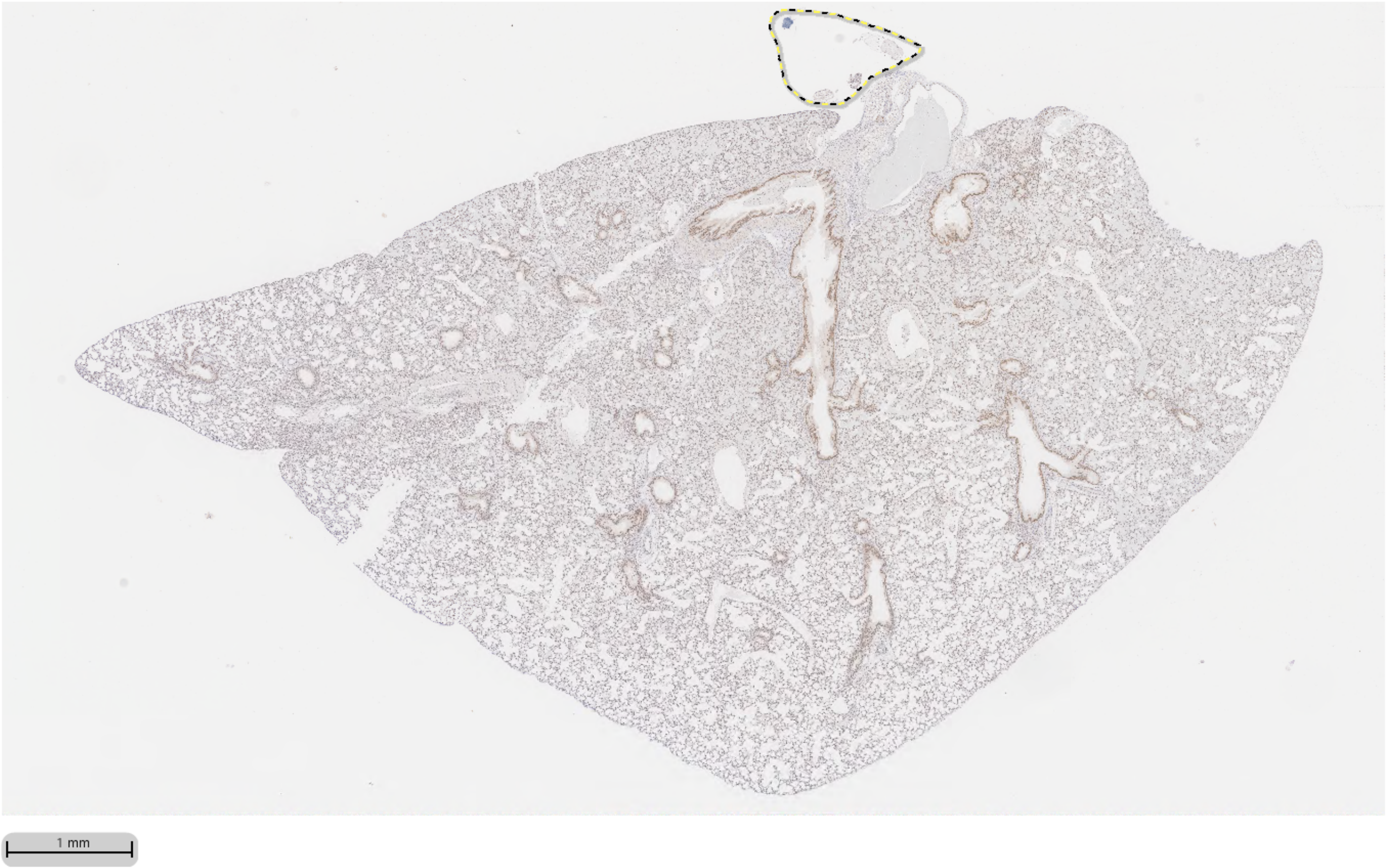

220524_3.quadriceps

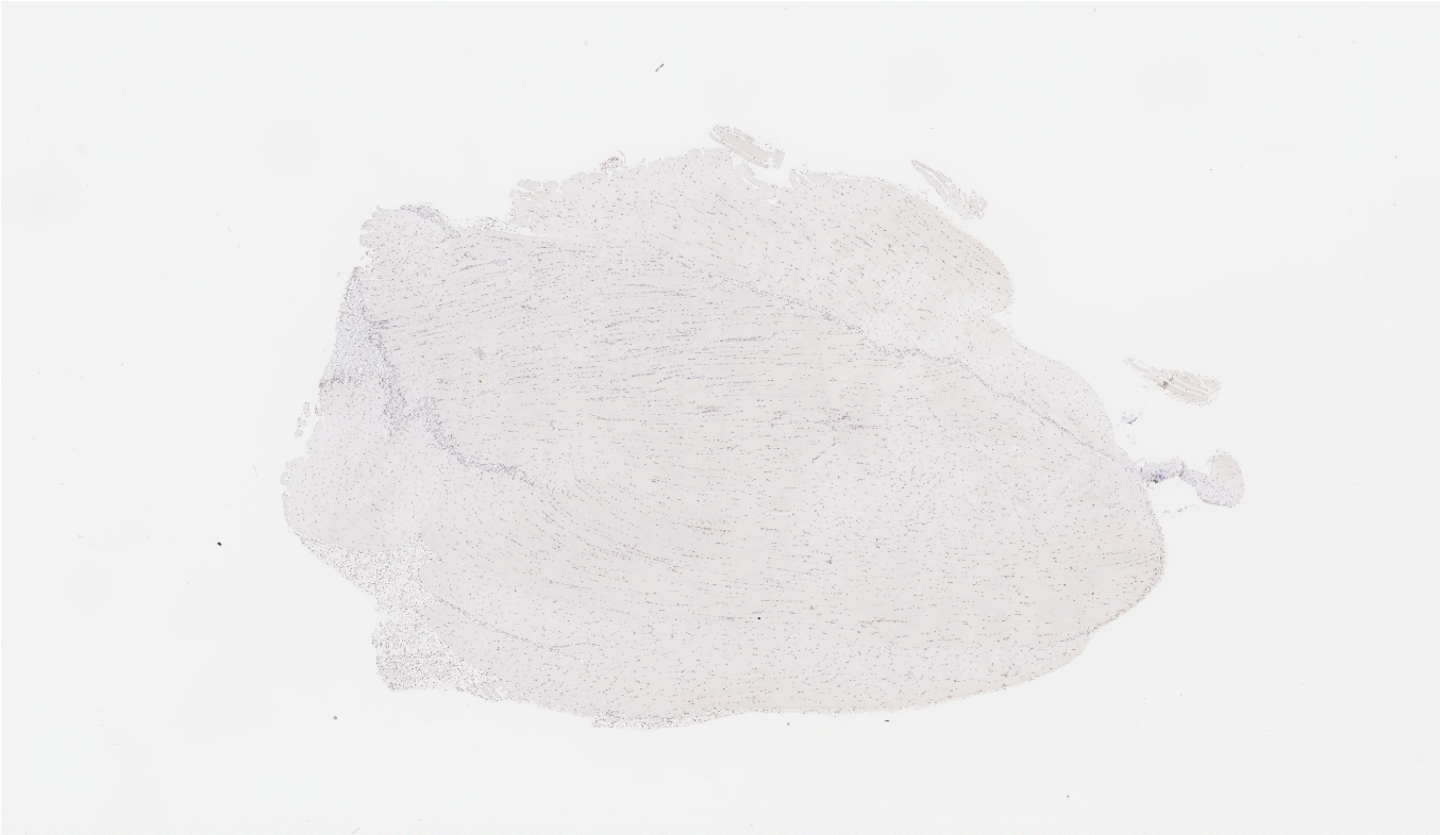

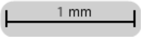

220524_4.brain

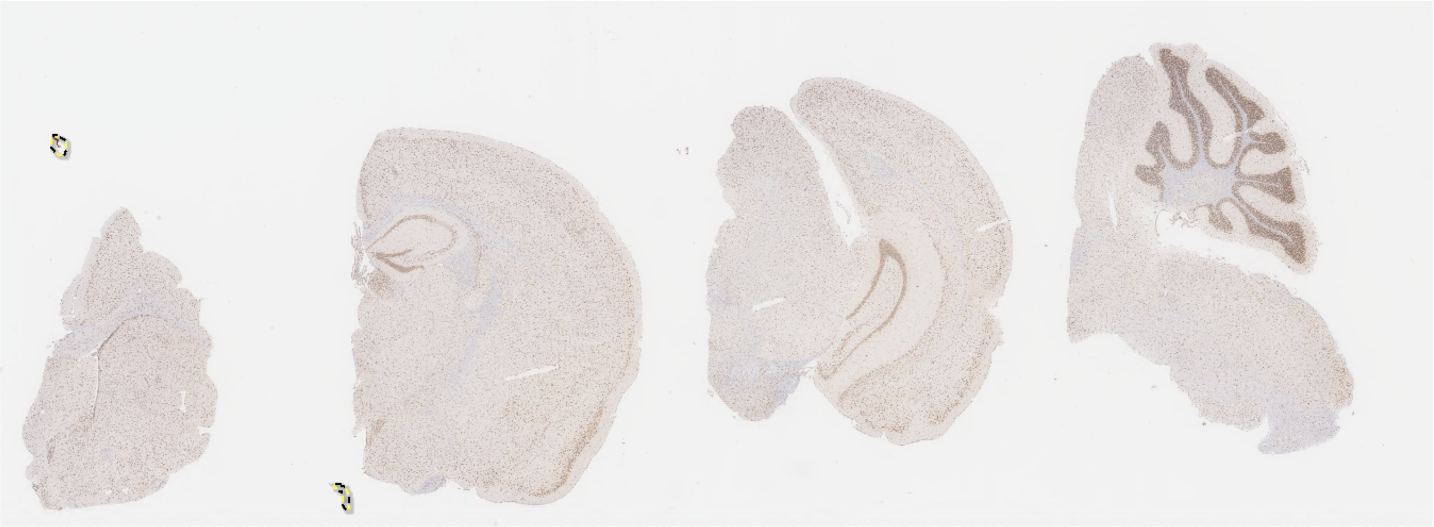

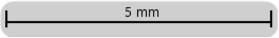

220524_4.lung

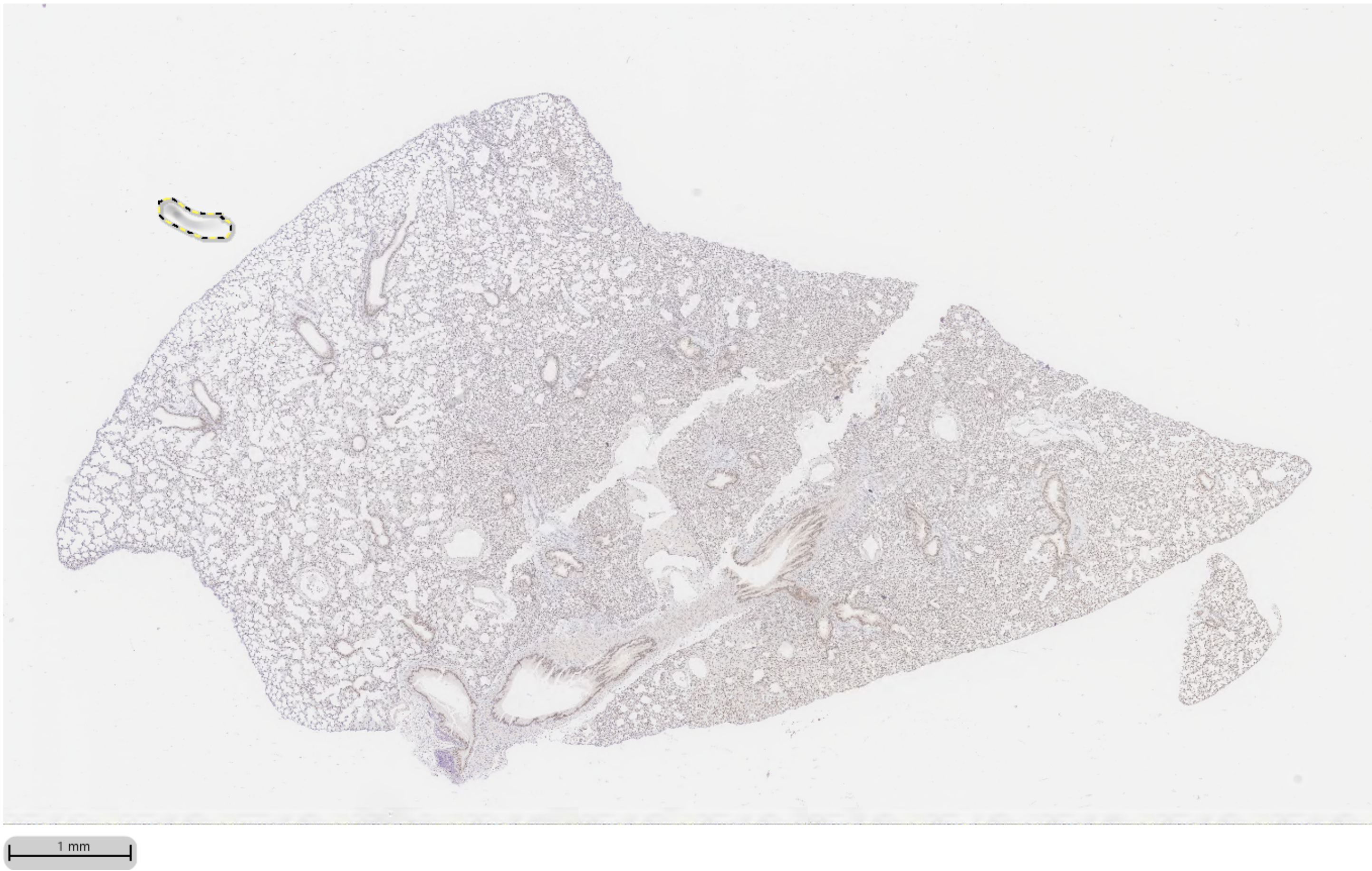

220524_4.quadriceps

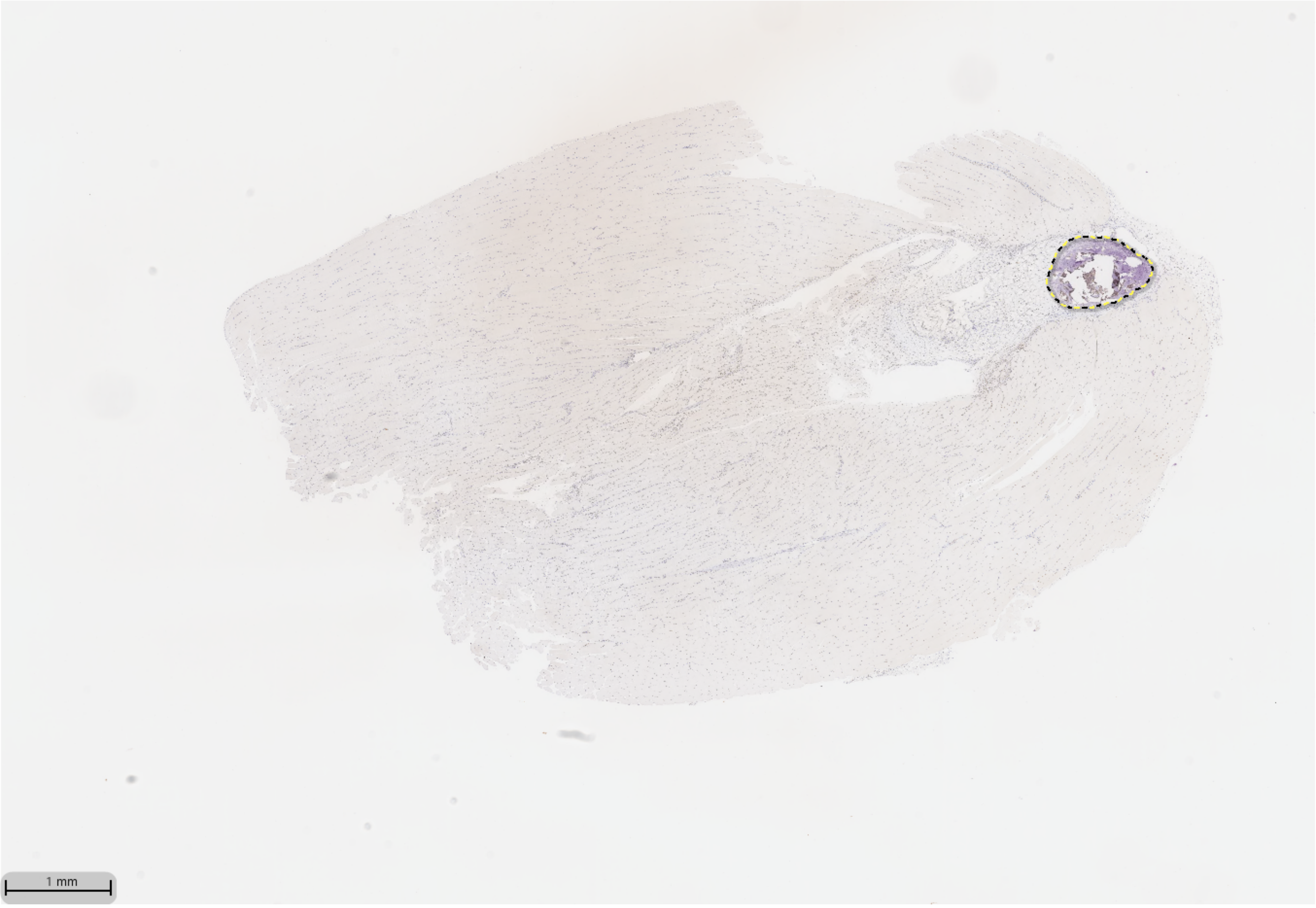

Supplement: S1 Appendix — (PDF) [file pgen.1011363.s025.pdf]
